# Supplementary material for: Two-dimensional and high-order directional information modulations for secure communications based on programmable metasurface
Source: Nat Commun. 2024 Jul 20;15:6140. doi: 10.1038/s41467-024-50482-y (PMC11271469; doi:10.1038/s41467-024-50482-y)
Supplement: Supplementary file 1 — Supplementary Information [file 41467_2024_50482_MOESM1_ESM.pdf]

Supplementary Information for

Two-dimensional and high-order directional information modulations for secure communications based on programmable metasurface

The Supplementary information includes:

- **Note 1. Comparison with the existing schemes**
- **Note 2. Demonstration of the directional security against harmonics**
- **Note 3. Demonstrating that our scheme can serve as a transmitter or receiver**
- **Note 4. The reference constellation diagram encoded with Gray code**
- **Note 5. Details of the optimization algorithm and the complexity analysis**
- **Note 6. Simulations for demonstrating the feasibility of the proposed method and comparing it with other existing algorithms**
- **Note 7. Process of the measuring signals**
- **Note 8. Measurements of the four-channel modulations**
- **Note 9. The magnitudes and phases of the measured fields for experiments**
- **Note 10. The distorted constellation diagrams in other directions**
- **Note 11. The experiment for measuring the direction of desired users**
- **Note 12. The transmission rate of the DIM scheme**
- **Note 13. The analysis of the secure zone**

Note 1. Comparison with the existing schemes

The comparison of our work in contrast to other existing categories achieving physical layer security (PLS) is listed in terms of directional security, system complexity, energy per bit, performance, realization strategy, advantages, and disadvantages, as summarized in Table S1. Specifically, we do not give the relevant performance comparison since the beamforming-based techniques and metasurface-coated devices are unable to achieve directional security. Compared with other existing schemes, our proposal has the following advantages:

- Our proposal can achieve two-dimensional and multi-channel (up to four channels and even more with larger-scale metasurfaces) DIM.
- Our proposal can achieve different high-order modulations, such as 8PSK, 16QAM, and 64QAM.
- Our proposal has the minimum energy per bit, being power-efficient.
- Our proposal is secure against harmonic compared with time-coding digital metasurfaces.
- Our proposal can serve as a low-profile transmitter or receiver, which greatly reduces system complexity.

1.1 Beamforming-based techniques

Beamforming (BF) based techniques have been widely employed for the PLS of wireless communications. The techniques are designed either to enhance the power at the receiver or to degrade the wiretap channel used by the eavesdropper. Additionally, BF often adopts other PLS techniques involving the hybridization of four methods[1-3] (1) secure BF, (2) artificial noise BF, (3) cooperative jamming-aided BF, and (4) joint channel coding with BF.

In summary, these methods increase the SNR difference between receiver and eavesdropper and they are motivated by Wyner's wiretap model. However, the information is still encoded in the time domain. The broadcast nature of wireless communication channels allows the signals in all directions to have an identical structure, only with different power levels. Therefore, an eavesdropper equipped with a sensitive receiver can intercept the information in theory. As a result, the demand for information security necessitates the idea of directional communications.

1.2 Metasurface-coated devices

The passive metasurface-coated devices can concentrate the power of ambient signals on the transmitter or receiver, which hopefully realizes energy efficiency, energy harvesting, and ultralow-power transmissions[4,5]. Furthermore, the physical layer security is ensured by increasing the power disparity between the receiver with the metasurface-coated device and scattered eavesdroppers. The device mitigates the critical problem of the tradeoff between ultralow-power transmissions and secrecy capacity for the first time, which is a great improvement compared with traditional beamforming techniques. However, although the power received by eavesdroppers is very low, the signal structure is still undistorted. Therefore, the security risk of the above method exists when an eavesdropper equipped with a sensitive receiver can theoretically intercept the information. Furthermore, the metasurface-coated device is passive, thus it is unable to realize real-time spatial wave manipulations and directional information modulation.

1.3 Phased arrays

The phased arrays usually require expensive transmitter and receiver (T/R) components for each radio-frequency channel, which results in a bulky and energy-hungry system. In the first realization of DIM based on phased arrays, the single-channel mode was realized using the QPSK modulation[6]. The phase shifts of each element are optimized based on a genetic algorithm to minimize the bit error rate (BER) of the desired direction while maximizing the BER elsewhere. The system employs four antenna elements, each equipped with a digital phase shifter of MITEQ. The total consumption is about 16 W, therefore the required energy per bit is 8 W/bit.

However, the system complexity is high, and it is hard to extend larger scales due to the expensive T/R components.

1.4 Time-domain coding metasurfaces

The time-domain coding metasurfaces (TDCM) integrate the electronically controllable

components, such as the varactor or positive-intrinsic-negative (PIN) diodes, with the low-cost metal patches. The realization of high-order modulations is to optimize the periodic sequences to change the magnitude and phase of the harmonic wave, respectively. Therefore, the TDCM-based DIM scheme is low-cost and power-efficient, and the complexity mainly depends on the optimization of the periodic sequences. However, the TDCM employs the harmonic of the electromagnetic (EM) waves, which will result in spectra pollution and is unable to ensure directional secure transmission due to the lack of space coding.

We here present two representative works about the TDCM-based DIM scheme. A single-channel 16QAM mode is implemented based on a varactor-loaded metasurface with 8×16 elements[7]. The phases and magnitudes of the harmonic signals are changed by the delay time and the relative phase difference of the pulse wave. The second example realizes the single-channel 256QAM transmission based on a 1-bit metasurface[8]. The metasurface consists of 56×20 meta-atoms, each equipped with a PIN diode of MADP-00097-14020. The total consumption is about 14.9 W, therefore the energy per bit is about 1.86 W/bit. The phases and magnitudes of the harmonic signals are changed by the introduction of delay time and duty ratio of the pulse wave. From the above examples, we find that the harmonic signal of arbitrary modulation can be realized by elaborately changing the form of the pulse wave in theory. However, the corresponding changes also occur at other-order harmonic at the same time, which means there exists the risk of information leakage.

1.5 Space-time-domain coding metasurfaces

Space-time-domain coding metasurfaces (STDCM) have a similar structure to TDCM, but the coding strategy is different. Firstly, the STDCM-based DIM scheme does not utilize periodic sequences, which means that the system does not generate the harmonic. We also prove that the received signals in the harmonic are noise. Secondly, the STDCM-based DIM scheme supports the manipulation of multiple spatial beams and temporal information with directional security.

We here present two representative examples that utilize the STDCM-based DIM scheme. The hardware is a 1-bit programmable metasurface with different scales and the method is a

modified Gerchberg-Saxton (GS) algorithm[9,10]. The demonstrations in Refs. [9] and [10] reported the dual-channel and three-channel QPSK modulations based on the STDCM, and the metasurfaces in Refs. [9] and [10] consist of 20×20 and 24×32 meta-atoms, respectively, each equipped with a PIN diode of MADP-00097-14020. The total consumption of their metasurfaces is 5.32 W and 10.2 W, and therefore the energy per bit is 1.33 W/bit and 1.7 W/bit.

However, these implementations are facing serval limitations. First, they mainly transmit signals. This is incompatible with the operation of traditional wireless systems, where the base station or terminal device can both transmit and receive information. Second, the metasurface needs an external feed horn, which causes several problems. It has a low energy conversion efficiency in case the feed source is located at a certain distance from the metasurfaces, capturing and modulating only part of the waves launched by the source. The freedom of the meta-atoms at the edge of the metasurfaces is wasted when the source only illuminates the central part of the transmissive metasurface to avoid the influence of diffraction. Meanwhile, the system has the occlusion effect caused by the feed source, which creates a partial blind spot. Finally, the high profile of the configuration composed of STDCM and the extended antenna is inconvenient for space-constrained applications such as satellites, vehicles, and aircraft. Moreover, the modulation is only BPSK or QPSK, which suffer from a lack of high-order modulation and QAM schemes that carry more information capacities.

1.6 The proposed DIM scheme

We demonstrated the four-channel 8PSK, 16QAM, and 64 QAM DIMs based on the scheme of STDCM. We designed and fabricated a low-profile programmable metasurface (PM), which supports the abilities of information modulation and radiation of EM waves. Therefore, the hardware overcomes the problems caused by the conventional metasurfaces. Furthermore, the hardware has a smaller number of meta-atoms (64) and a higher phase quantization (2-bit), as compared with Refs. [9] and [10]. Although the scale is not as large as that in the previous work, our system has more degrees of freedom (DoF). We further develop a fast and feasible algorithm to efficiently employ the massive number of DoFs. We have also compared it with other methods reported in the field of DIM, such as the GA, modified GS, and squared-infinity norm

Douglas-Rachford splitting. The simulations demonstrate that our method has faster convergence, better performance, and scalability.

The PM consists of 8×8 meta-atoms, each equipped with four PIN diodes of MADP-000907-14020. The total power consumption of the metasurface is 2.55 W. The current system currently supports up to four channels of 64QAM transmissions, which is equivalent to $4\times6=24$ bits of information at a time. Therefore, the energy per bit of our design is about 0.106 W/bit, which is the smallest among all the reported systems.

Table S1. Comparison with reported schemes achieving physical-layer security

	**Directional security**	**System Complexity**	**Energy/bit**	**Performance**	**Realization strategy**	**Advantages**	**Disadvantages**
**Beamforming-based techniques**	**No**	--		--	--	--	--
**Metasurface-coated devices**	**No**	--		--	--	--	--
**Phased arrays**	**Yes**	**High:** the utilization of expensive T/R components	8 W	Single-channel QPSK modulation[6]	A four-element linear array; GA algorithm	The continuous phase shifts	Poor scalability due to expensive cost
**Time-domain coding metasurfaces**	**No:** Information leakage on harmonics	**Low:** optimization of periodic sequences	--	Single-channel 16QAM[7]	A varactor-loaded metasurface with 8×16 elements; Optimization of periodic waves	The continuous phase shifts and magnitudes;	Unsecure on harmonic; Single-channel mode;
			1.86 W	Single-channel 256QAM[8]	A 1-bit metasurface with 56×20 elements; Optimization of periodic waves		
**Time-space-domain coding metasurfaces**	**Yes**	**Low:** optimization of coding sequences	1.33 W	Dual-channel QPSK modulation[9]	A 1-bit metasurface with 20×20 elements;	Secure against harmonic; Two-dimensional	Finite phase shifts; Low-order modulation;

					Modified GS algorithm	and multiple-channel mode.	
			0.34 W	Three-channel BPSK modulation[10]	A 1-bit metasurface with 24×32 elements; Modified GS algorithm		
**Our work**	**Yes**	**Low:** optimization of coding sequences	**0.106 W**	Four-channel 8PSK, 16QAM and 64QAM	A 2-bit metasurface with 8×8 elements; ADMM-based algorithm	Secure against harmonic; Two-dimensional and multiple-channel mode; Serve as transmitter or receiver with low profile; High-order modulation;	Finite phase shifts; Small-scale array

Note 2. Demonstration of the directional security against harmonics

Our DIM scheme utilizes a low-profile programmable metasurface and changes the coding sequence in real-time, which corresponds to a control sequence with infinitely long random periods. According to the Fourier theory, the frequency offset of harmonics is zero, thus ensuring that our scheme is secure against harmonics.

Proof: The received signal at moment *t* is expressed as,

$$E(\theta_k,\ \varphi_k;\ t) = e^{j2\pi f_0 t}\sum_{p=1}^{P}\sum_{q=1}^{Q}\sqrt{G(\theta_k,\ \varphi_k)}x_{p,q}(t)e^{j\mathbf{v}_{p,q}^{H}\mathbf{u}} + n_k, \tag{S1}$$

where f_0, θ_k, φ_k, $G(\theta_k,\ \varphi_k)$, $x_{p,q}$, and n_k are the central frequency, the elevation angle, the azimuth angle, the directional gain, the optimized phase response of the element, and the noise with respect to the *k*th user, respectively. In addition, $\mathbf{v}_{p,q} = [pd_x,\ qd_y]^T$ and

$\mathbf{u} = 2\pi/\lambda[\sin\theta_k \cos\varphi_k,\ \sin\theta_k \sin\varphi_k]^T$ are the auxiliary vectors to simplify Equation (S1).

For the *K*-bit phase quantization, the response $x_{p,q}(t)$ at moment *t* is expressed as

$$x_{p,q}(t) = \sum_{i=0}^{\infty} a_{p,q}^{i} \Gamma(t - iT), \tag{S2}$$

where $a_{p,q}^{i} \in \{1/\sqrt{N}\, e^{jw_m} \mid w_i = 2\pi \cdot m/2^K,\ m = 1,\ 2,\ ...,\ 2^K\}$ is the optimized discrete response of the (*p*, *q*) element for generating the desired field in the target direction. The optimized coding sequences are first stored in the FPGA and switched every passing *T* gap. The rectangular pulse signal $\Gamma(t)$ is

$$\Gamma(t) = \begin{cases} 1, & 0 \le t \le T \\ 0, & otherwise \end{cases}. \tag{S3}$$

We perform the Fourier transform of Equation (S1) and obtain the spectral response of the received signal, namely,

$$E(\theta_k,\ \varphi_k; f) = \sum_{p=1}^{P} \sum_{q=1}^{Q} \sqrt{G(\theta_k,\ \varphi_k)}\, x_{p,q}(f - f_0) e^{j\mathbf{v}_{p,q}^{H}\mathbf{u}} + n_k(f). \tag{S4}$$

In the above equation, the spectral response of the response $x_{p,q}(t)$ is

$$x_{p,q}(f) = \sum_{i=0}^{\infty} a_{p,q}^{i} \Gamma(f) e^{-j2\pi f i T}, \tag{S5}$$

where the spectral response of the pulse signal $\Gamma(t)$ is

$$\Gamma(f) = \int_{-\infty}^{\infty} \Gamma(t) e^{-j2\pi f t} dt = \frac{\sin(\pi f T)}{\pi f}. \tag{S6}$$

It is noticed that the spectral response of the received signal at the harmonic frequency $f_0 + nf_T$ ($f_T = 1/T$) is

$$
\begin{aligned}
E(\theta_k,\ \varphi_k; f_0+nf_T) &= \sum_{p=1}^{P}\sum_{q=1}^{Q}\sqrt{G(\theta_k,\ \varphi_k)}x_{p,q}(nf_T)e^{j\boldsymbol{v}_{p,q}^{H}\boldsymbol{u}} + n_k(f+nf_T) \\
&= \sum_{p=1}^{P}\sum_{q=1}^{Q}\sqrt{G(\theta_k,\ \varphi_k)}e^{j\boldsymbol{v}_{p,q}^{H}\boldsymbol{u}}\sum_{i=0}^{\infty}a_{p,q}^{i}\Gamma(nf_T)e^{-j2\pi nif_T T} \\
&\quad + n_k(f+nf_T) \qquad\qquad , \ n \neq 0. \qquad \text{(S7)} \\
&= \sum_{p=1}^{P}\sum_{q=1}^{Q}\sqrt{G(\theta_k,\ \varphi_k)}e^{j\boldsymbol{v}_{p,q}^{H}\boldsymbol{u}}\sum_{i=0}^{\infty}a_{p,q}^{i}\frac{\sin(n\pi f_T T)}{n\pi f_T}e^{-j2\pi nif_T T} \\
&\quad + n_k(f+nf_T) \\
&= n_k(f+nf_T)
\end{aligned}
$$

It is worth emphasizing that the value of the harmonic frequency is determined according to the definition of the time-modulated array or time-coding metasurface[11]. Equation(S7) indicates that the strength of the received signal in the target direction at the harmonic frequency $f_0 + nf_T$ is the noise, which demonstrates that our proposal is secure transmission against harmonic.

To demonstrate the unique secure feature against harmonic, we perform simulations of the dual-channel 8PSK configuration. The two users are located at $(\theta,\ \varphi) = (40°, 0°)$ and $(\theta,\ \varphi) = (25°, 90°)$, respectively. The switching time of FPGA is 10 us (i.e., the frequency offset is $f_T = 100$ KHz in according with the above definition) and the signal length is 5 ms. The received signals are calculated using Equation (S4) and normalized, and the SNR is 15 dB.

As shown in Figure S1, the received signals of user 1 at the central frequency f_0 have the characteristic of the 8PSK symbols, in which the magnitudes fluctuate around 1 and the phases are distributed in the vicinity of the eight discrete values (i.e., -180˚, -135˚, -90˚, -45˚, 0˚, 45˚, 90˚, and 135˚). The signals received by user 2 are demonstrated in Figure S2, in which the magnitude values are relatively lower than those of user 1 and fluctuate between 0.6~1. However, the phase values have a form similar to the 8PSK symbols, which implies that the fluctuations of magnitude do not affect the signal recovery. Furthermore, we present the signals received by user 1 at harmonic $f_0 + f_T$ and $f_0 + 2f_T$ in Figures S3a and S3b, respectively,

where the magnitude values are low and the phase distributions are disorganized. The above results demonstrate that the received signals at harmonic are mainly the noise and the information is unable to be transmitted.

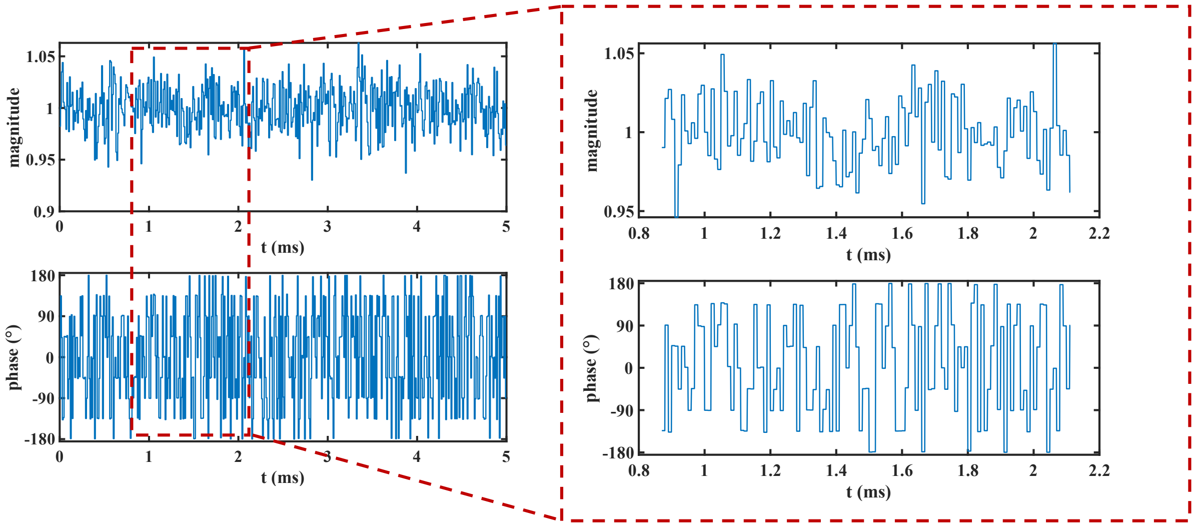

Figure S1. The signals received by user 1 with about 5 ms. User 1 is located at $(\theta, \varphi) = (40°, 0°)$ and the frequency is f_0. The signals within 0.85-2.15 ms are enlarged on the right.

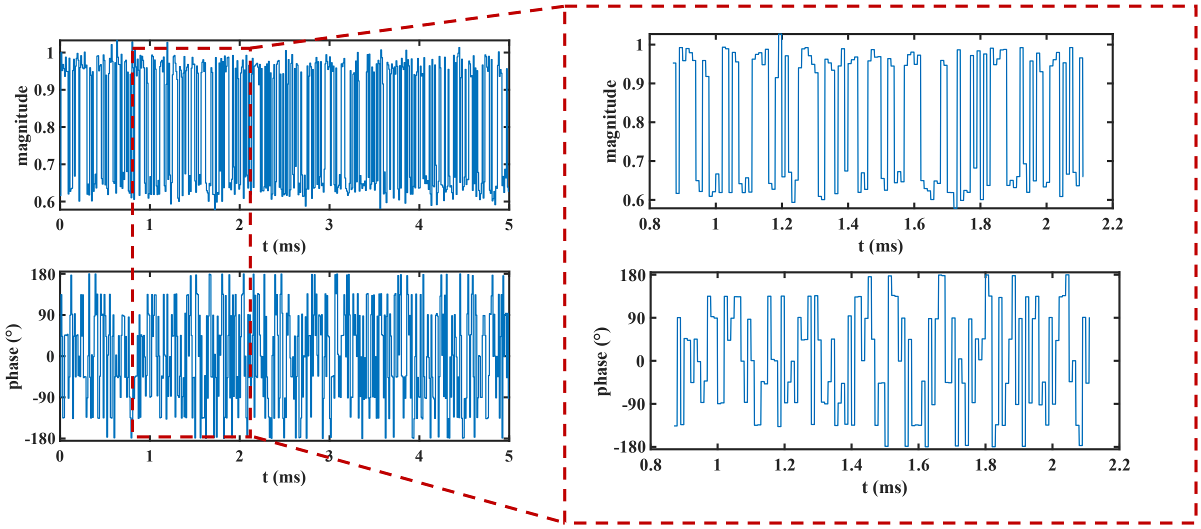

Figure S2. The signals received by user 2 with about 5 ms. User 2 is located at $(\theta, \varphi) = (25°, 90°)$ and the frequency is f_0. The signals within 0.85-2.15 ms are enlarged on the right.

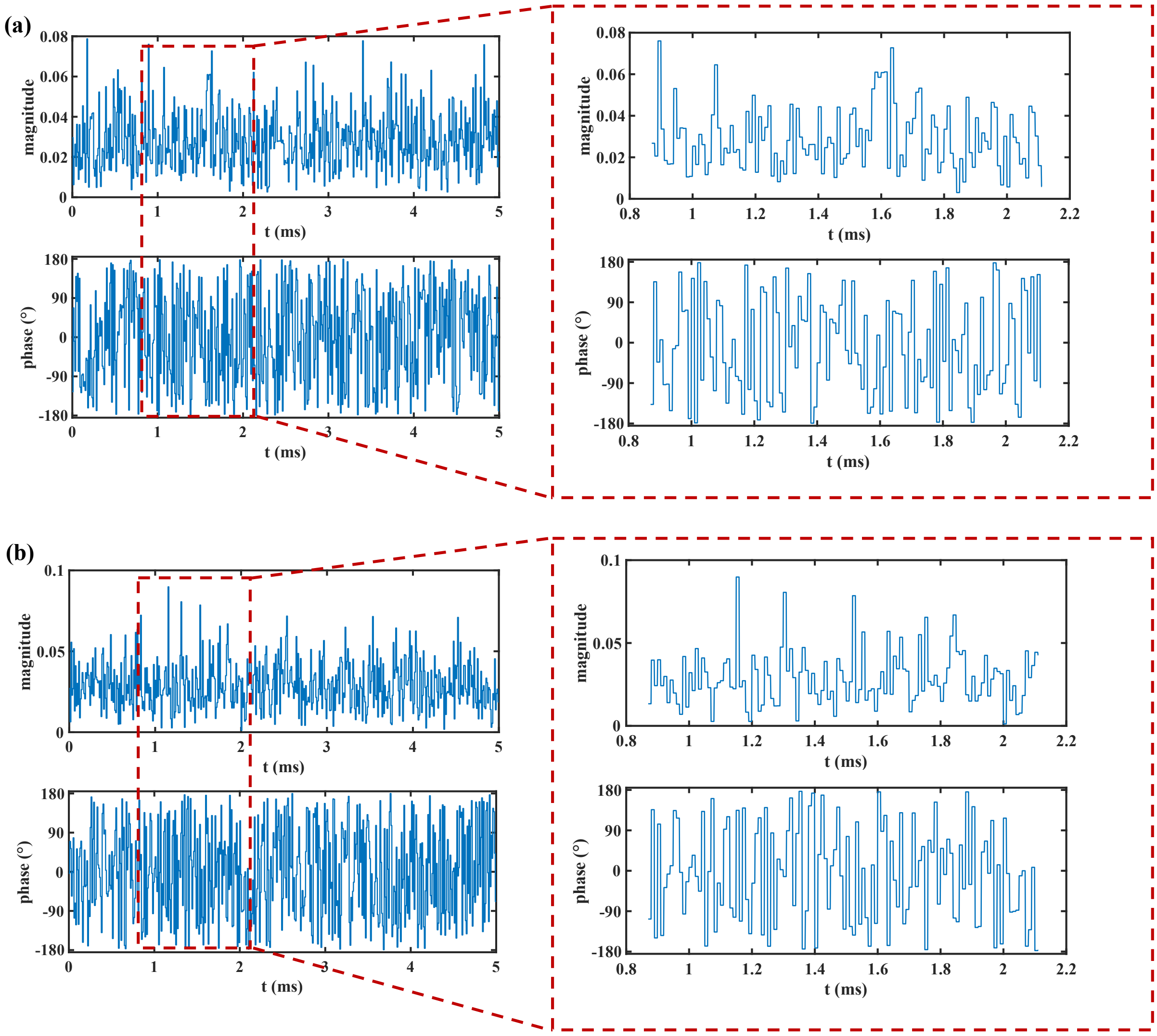

Figure S3. (**a**) The signals received by user 1 within 5 ms and the signal frequency is $f_0 + f_T$. Further, the signals within 0.85-2.15 ms are enlarged on the right. (**b**) The signals at harmonic $f_0 + 2f_T$.

Note 3. Demonstrating that our scheme can serve as a transmitter or receiver

Our hardware design integrates the advantages of programmable metasurfaces and phased array antennas. To demonstrate the unique property, we experimented with transmitting and receiving signals using the same programmable metasurface. Figure S4 illustrates the experimental scene, in which the transmitter and receiver are equipped with the identical device. In the experiment, the transmitter radiates 8PSK constellation symbols to the desired direction $(\theta, \varphi) = (-17°, 0°)$. Figure S5a presents the magnitudes of measured fields for the single-channel 8PSK mode, in which the main lobe beams are located near the target direction

$\theta = -17°$, and the normalized radiation powers are larger than 0.8. As illustrated in Figure S5b, the phases of measured fields have an interval of nearly 45° in the target direction. The above results demonstrate that the single-channel 8PSK modulation is realized. We here should admit that the performance of the measurements is relatively poor compared to the case of utilizing a high-gain horn antenna as a transmitter. However, the problem can easily be mitigated by increasing the array scale.

We observe that the error vector magnitude (EVM) values in the vicinity of the target directions are relatively lower. At the bottom of Figure S6, the constellation diagrams in different directions are presented. The diagram in the target direction matches well with the reference, while the others are distorted, which validates the security property of the proposed method. The above measurements demonstrate that DIM can be implemented by exploiting the programmable metasurfaces as the transmitter and receiver.

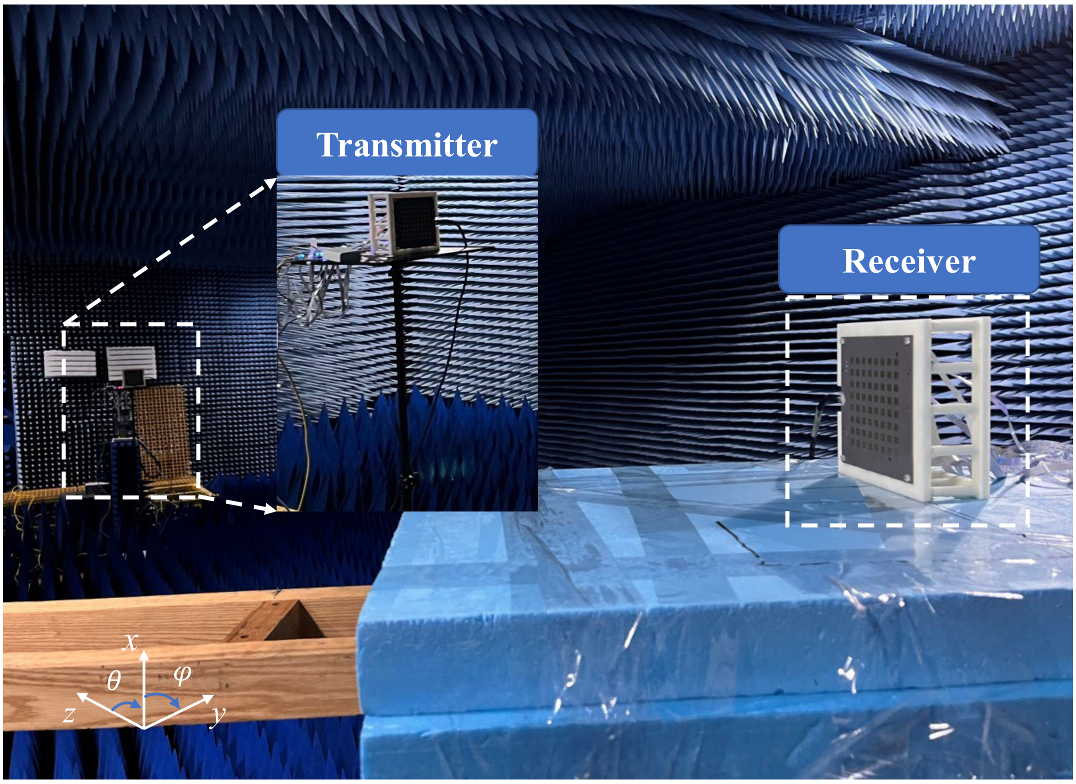

Figure S4. Experimental scene for validating that our scheme can serve as a transmitter or receiver.

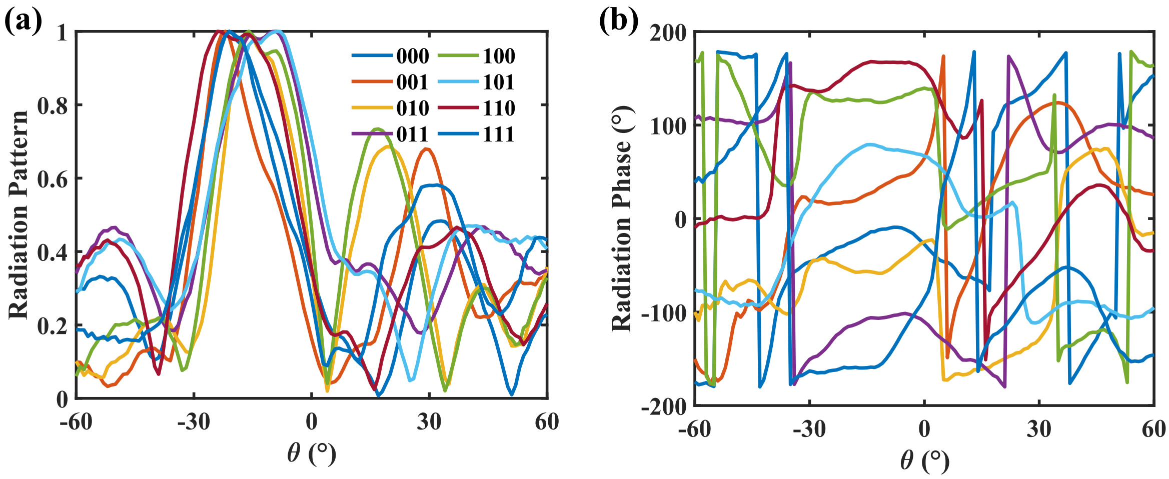

Figure S5. The measured fields for the 8PSK scheme where the transmitter and receiver are implemented using the same programmable metasurface. (**a**) The magnitudes of measured fields. (**b**) The phases of measured fields.

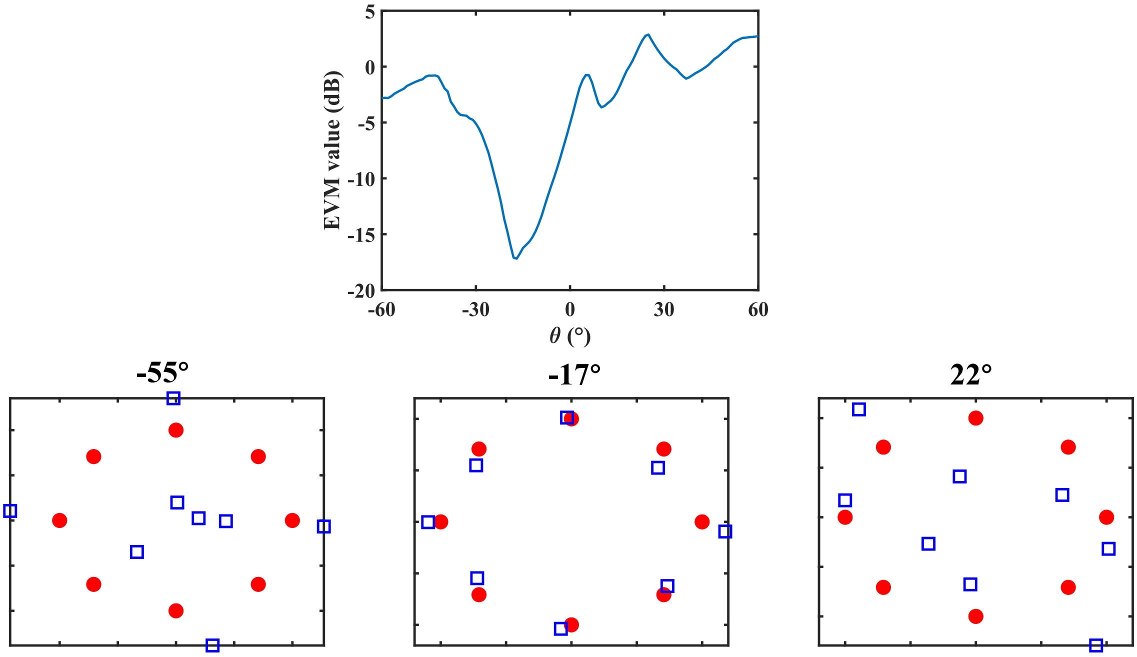

Figure S6. The EVM for validating the security of DIM for the 8PSK scheme where the transmitter and receiver are implemented using the same programmable metasurface. The bottom presents three constellation diagrams at different elevation angles.

Note 4. The reference constellation diagram encoded with Gray code

The received signals is most likely to be interpreted as its neighboring signal due the measurement noise and the uncertainty of the system model. To reduce the overall bit error rate

(BER), the constellation symbols are encoded by Gray code. Specifically, the digital bits of adjacent symbols differ by only one bit, as illustrated in Figure S7.

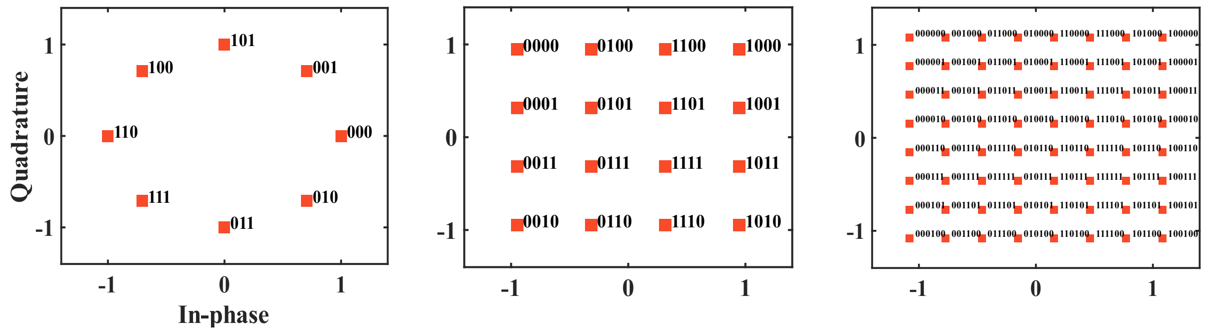

Figure S7. The reference constellation diagrams of 8PSK, 16QAM, and 64QAM schemes. Each symbol point is encoded with the Gray code to improve the noise immunity.

Note 5. Details of the optimization algorithm and the complexity analysis

S5.1 Details of the optimization algorithm

1) The update strategy of $\tilde{\mathbf{x}}^{\mathrm{t}}$

By fixing the variables $\mathbf{x}^t$, β^t, $\mathbf{u}^t$ and ignoring the constant terms, we rewrite the problem (6a) as

$$\begin{aligned}
\tilde{\mathbf{x}}^{t+1} &= \arg\max_{\tilde{\mathbf{x}}} \quad \mathcal{L}(\mathbf{x}^t,\ \beta^t,\ \tilde{\mathbf{x}},\ \mathbf{u}^t), \\
&= \arg\max_{\tilde{\mathbf{x}}} \quad \left\|\mathbf{s}-\beta^t\mathbf{H}\mathbf{x}^t\right\|_2^2 + K(\beta^t)^t\sigma^2 + \Re\{(\mathbf{u}^t)^H(\tilde{\mathbf{x}}-\mathbf{x}^t)\} + \frac{1}{2}\rho\left\|\tilde{\mathbf{x}}-\mathbf{x}^t\right\|_2^2, \\
&= \arg\max_{\tilde{\mathbf{x}}} \quad \Re\{(\mathbf{u}^t)^H\tilde{\mathbf{x}}\} + \frac{1}{2}\rho\left\|\tilde{\mathbf{x}}-\mathbf{x}^t\right\|_2^2, \\
&= \arg\max_{\tilde{\mathbf{x}}} \quad \Re\{(\mathbf{u}^t)^H\tilde{\mathbf{x}}\} - \rho\Re\{(\mathbf{x}^t)^H\tilde{\mathbf{x}}\}, \\
&= \arg\max_{\tilde{\mathbf{x}}} \quad \Re\{(\tilde{\boldsymbol{\phi}}^t)^H\tilde{\mathbf{x}}\},
\end{aligned} \tag{S8}$$

where $\tilde{\boldsymbol{\phi}}^t = \mathbf{u}^t - \varrho\mathbf{x}^t$. Then the problem (6a) is transformed as

$$\begin{aligned}
\min_{\tilde{\mathbf{x}}} \quad & \Re\{(\tilde{\boldsymbol{\phi}}^t)^H\tilde{\mathbf{x}}\} \\
\text{s. t.} \quad & \tilde{\mathbf{x}}^{t+1}(i) \in \boldsymbol{\mathcal{X}},\ i=1,\ \ldots,N
\end{aligned}. \tag{S9}$$

Note that the goal function and the constraint are separable, which means that the problem can be converted into the N parallel single variate optimizations, namely,

$$\min_{w_i^{t+1}} \quad \cos(w_i^{t+1} - \alpha_i^t)$$
$$\text{s. t.} \quad \frac{1}{\sqrt{N}} e^{jw_i^{t+1}} \in \boldsymbol{\mathcal{X}},\ i = 1,\ ...,\ N \quad , \tag{S10}$$

where w_i^{t+1} and α_i^t are the phase of $\tilde{\mathbf{x}}^{t+1}(i)$ and $\tilde{\boldsymbol{\phi}}^t$, respectively. By exhaustively searching $\boldsymbol{\mathcal{X}}$, the closed solution is $w_i^{t+1} = l\frac{2\pi}{2^P}$, where P is the quantization level, and the optimal index l is

$$l = \arg\min_{l=1,\ ...,\ 2^P} \ \{\tau_l\}, \tag{S11}$$

where $\tau_l = \cos(l\frac{2\pi}{2^P} - \alpha_i^t)$.

2) The update strategy of $\mathbf{x}^t$

By fixing the variables $\tilde{\mathbf{x}}^{t+1}$, β^t, $\mathbf{u}^t$ and ignoring the constant terms, we rewrite the problem (6b) as

$$\begin{aligned}
\mathbf{x}^{t+1} &= \arg\max_{\mathbf{x}} \quad \mathcal{L}(\mathbf{x},\ \beta^t,\ \tilde{\mathbf{x}}^t,\ \mathbf{u}^t), \\
&= \arg\max_{\mathbf{x}} \quad \left\|\mathbf{s} - \beta^t\mathbf{H}\mathbf{x}\right\|_2^2 + K(\beta^t)^2\sigma^2 + \Re\{(\mathbf{u}^t)^H(\tilde{\mathbf{x}}^{t+1} - \mathbf{x})\} + \frac{1}{2}\rho\left\|\tilde{\mathbf{x}}^{t+1} - \mathbf{x}\right\|_2^2, \\
&= \arg\max_{\mathbf{x}} \quad \left\|\mathbf{s} - \beta^t\mathbf{H}\mathbf{x}\right\|_2^2 - \Re\{(\mathbf{u}^t)^H\mathbf{x}\} - \rho\Re\{(\tilde{\mathbf{x}}^{t+1})^H\mathbf{x}\} + \frac{1}{2}\rho\mathbf{x}^H\mathbf{x}, \\
&= \arg\max_{\mathbf{x}} \quad \mathbf{x}^H((\beta^t)^2\mathbf{H}^H\mathbf{H} + \frac{\rho}{2}\mathbf{I}_N)\mathbf{x} - \Re\{(2\beta^t\mathbf{H}^H\mathbf{s} + \mathbf{u}^t + \rho\tilde{\mathbf{x}}^{t+1})^H\mathbf{x}\}, \\
&= \arg\max_{\mathbf{x}} \quad \mathbf{x}^H\mathbf{R}_H\mathbf{x} - \Re\{(\boldsymbol{\phi}^{t+1})^H\mathbf{x}\},
\end{aligned} \tag{S12}$$

where $\mathbf{R}_H = (\beta^t)^2\mathbf{H}^H\mathbf{H} + \frac{\varrho}{2}\mathbf{I}_N$ and $\boldsymbol{\phi}^{t+1} = 2\beta^t\mathbf{H}^H\mathbf{s} + \mathbf{u}^t + \varrho\tilde{\mathbf{x}}^{t+1}$. The problem then becomes

$$\min_{\mathbf{x}} \quad \mathbf{x}^H\mathbf{R}_H\mathbf{x} - \Re\{(\boldsymbol{\phi}^{t+1})^H\mathbf{x}\}. \tag{S13}$$

By setting the differential of the objective function with respect to $\mathbf{x}$ as zero, we obtain $\nabla_{\mathbf{x}^*}[\mathbf{x}^H\mathbf{R}_H\mathbf{x} - \Re\{(\boldsymbol{\phi}^{t+1})^H\mathbf{x}\}] = \mathbf{R}_H\mathbf{x} - \frac{1}{2}\boldsymbol{\phi}^{t+1} = 0$.

Then we obtain

$$\mathbf{x}^{t+1} = \frac{1}{2}(\mathbf{R}_H)^{-1}\boldsymbol{\phi}^{t+1}. \tag{S14}$$

3) The update strategy of β^t

The closed form of β^{t+1} is expressed as follows with fixed $\tilde{\mathbf{x}}^{t+1}$, $\mathbf{x}^{t+1}$, $\mathbf{u}^t$, namely,

$$\begin{aligned}\beta^{t+1} &= \arg\max_{\beta>0} \quad \|\mathbf{s}-\beta\mathbf{H}\mathbf{x}\|_2^2 + K\beta^2\sigma^2, \\ &= \arg\max_{\beta>0} \quad -2\beta\Re\{\mathbf{s}^H\mathbf{H}\mathbf{x}^{t+1}\} + \beta^2\left\|\mathbf{H}\mathbf{x}^{t+1}\right\|_2^2 + \beta^2 K\sigma^2, \\ &= \frac{\Re\{\mathbf{s}^H\mathbf{H}\mathbf{x}^{t+1}\}}{\left\|\mathbf{H}\mathbf{x}^{t+1}\right\|_2^2 + K\sigma^2}.\end{aligned} \tag{S15}$$

We take the absolute value of the β^{t+1} to ensure that β^{t+1} is greater than zero, namely,

$$\beta^{t+1} = \left|\frac{\Re\{\mathbf{s}^H\mathbf{H}\mathbf{x}^{t+1}\}}{\left\|\mathbf{H}\mathbf{x}^{t+1}\right\|_2^2 + K\sigma^2}\right|. \tag{S16}$$

S5.2 The computational complexity

We analyze the computational complexity of the proposed discrete optimization algorithm in terms of the number of multiplication operations. The computational complexity of the optimization framework is mainly introduced by the updates of variables $\tilde{\mathbf{x}}^t$, $\mathbf{x}^t$, β^t, and $\mathbf{u}^t$. We calculate the complexity as follows. In the updates of $\tilde{\mathbf{x}}^t$, we convert the problem into N parallel univariate optimizations, and each subproblem is solved by exhaustively searching all discrete phases. Thus, the computational complexity is $\mathcal{O}(N \cdot 2^P)$. The updates of $\mathbf{x}^t$ mainly depend on the inverse operation of $\mathbf{R}_H$, and its complexity is $\mathcal{O}(N^3)$. The complexity of $(\mathbf{R}_H)^{-1} \cdot \boldsymbol{\phi}^t$ is $\mathcal{O}(N^2K)$. Thus, the computational complexity of the updates of $\mathbf{x}^t$ is $\mathcal{O}(N^3 + N^2K)$. Clearly, the computational complexity of β^t and $\mathbf{u}^t$ are $\mathcal{O}(NK)$ and $\mathcal{O}(N)$, respectively. In summary, the overall complexity of T iterations of the algorithm is $\mathcal{O}(T \cdot (N^3 + N^2K + N(K + 2^P)))$.

Note 6. Simulations for demonstrating the feasibility of the proposed method and comparing it with other existing algorithms

S6.1 Demonstration of the feasibility of the proposed method

To demonstrate the feasibility of the proposed ADMM-based method for various modulation schemes and channels, we conduct three representative simulation cases as summarized in Table S2. In Case I, the modulation scheme is 8PSK, and there are six desired directions. The

first three are along $\theta = 18°,\ 50°,\ \text{and}\ -30°$, respectively, on the $\varphi = 0°$ plane; while the last three are along $\theta = 33°,\ -11°,\ \text{and}\ -51°$, respectively, on the $\varphi = 45°$ plane. In Case II, the scheme is 16QAM with four desired directions. Specially, the first two are along $\theta = 20°$ and $-30°$, respectively, on the $\varphi = 0°$ plane; while the last two are along $\theta = 34°$ and $-41°$, respectively, on the $\varphi = 45°$ plane. In Case III, the scheme is 64QAM with four desired directions. Specially, the first two are along $\theta = 28°$ and $-31°$, respectively, on the $\varphi = 0°$ plane; while the last two are along $\theta = 41°$ and $-34°$, respectively, on the $\varphi = 90°$ plane. A special point is that the SNR and quantization level are varied in Cases II and III to verify the robustness of the algorithm. For each of the simulations, the symbols in different desired directions are selected randomly, and 5000 times of realization are performed in the Monte Carlo simulations.

Table S2. Simulation configurations for validating the scheme, see the explanations in the main text.

Scenario	Scheme	Desired directions	SNR (dB)	Quantization level (bits)
Case I	8PSK	$(\theta_1 = 18°,\ \theta_2 = 50°,\ \theta_3 = -30°,\ \varphi_{1,\,2,\,3} = 0°)$ $(\theta_4 = 33°,\ \theta_5 = -11°,\ \theta_6 = -51°,\ \varphi_{4,\,5,\,6} = 45°)$	10	2
Case II	16QAM	$(\theta_1 = 20°,\ \theta_2 = -30°,\ \varphi_{1,\,2} = 0°)$ $(\theta_3 = 34°, \theta_4 = -41°,\ \varphi_{3,\,4} = 45°)$	10, 20	2, 3, 4
Case III	64QAM	$(\theta_1 = 28°,\ \theta_2 = -31°,\ \varphi_{1,\,2} = 0°)$ $(\theta_3 = 41°,\ \theta_4 = -34°,\ \varphi_{3,\,4} = 90°)$	10, 20	2, 3, 4

Once the configuration is specified, the algorithm is executed to find the optimized coding sequences for each transmitted constellation symbol. In all simulations, the PM consists of 8×8 independent elements with half-wavelength spacing at the operating frequency of 11 GHz. The parameters of the discrete optimization algorithm are as follows: the initial coding sequences

$\boldsymbol{x}^{(0)}$, dual vector $\boldsymbol{u}^{(0)}$, penalty parameter ρ, and iteration number T are $1/\sqrt{N}[1, 1, ..., 1]^H$, $1/\sqrt{N}[1, 1, ..., 1]^H$, 1, and 100, respectively. The algorithm was coded in MATLAB and carried out on a computer with an Intel(R) Core (TM) i5-8300H CPU.

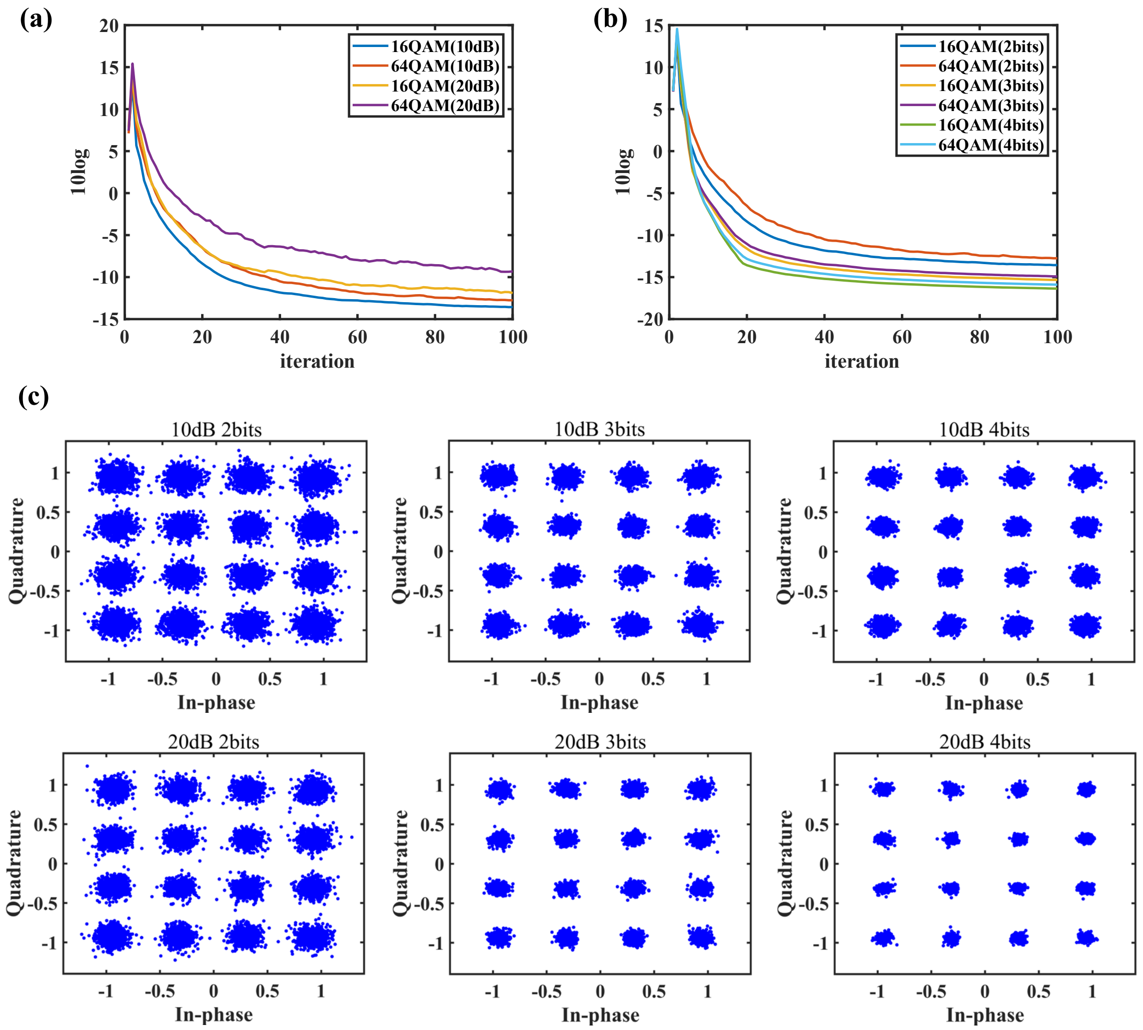

Figure S8. Simulations that validate the feasibility of the proposed discrete optimization algorithm. (**a**)-(**b**) Variations of the Lagrangian functions for Cases II and III. (**a**) The quantization level is fixed to 2 bits, while SNRs are 10 and 20 dB, respectively. (**b**) SNR is fixed to 10 dB, while the quantization levels are 2, 3, and 4 bits, respectively. (**c**) The received signals in all desired directions for Case II, in which the quantization level changes from 2 bits to 4 bits, while the SNR changes from 10 dB to 20 dB.

Figures S8a and S8b present the variations of the Lagrangian functions for Cases II and III. The curves exhibit similar behavior, namely, they first experience a short rise and then rapidly decline to stability, which exhibits the fast convergence and stable performance of the algorithm.

Moreover, Figure S8b indicates that increasing the quantization level of the element phase will reduce the Lagrangian values, and the reduction is slight when the quantization level increases from 2 to 4 bits. Considering the balance between performance and complexity, the 2-bit quantization is deemed sufficient. The received signals along four desired directions for Case II are illustrated in Figure S8c, where sixteen distinct clusters can be identified, being the characteristics of 16QAM. With the increment of SNR and quantization level, the clusters are more concentrated. Overall, these results validate the accuracy and feasibility of the proposed optimization algorithm.

To further quantitatively evaluate the correctness of the received signals, the bit error rate (BER) distributions are presented in Figure S9 as the functions of directions. The lowest BER values are concentrated in the desired directions, while the higher BER values are observed in other directions, thus showing the directional-security features. Specifically, the 8PSK scheme in Case I exhibits the lowest BER, which owns to its low coding capacity. Furthermore, the BER is relatively low in the vicinity of the desired directions for each case, which are referred to as the secure zones. The signals within the secure zones remain similar to the standard constellation diagrams, which increases the robustness of the communications when the user's directions deviate slightly. Nevertheless, it also brings security risks when an eavesdropper is close to or aligned with the user's directions. To mitigate this issue, increasing the array aperture or simultaneously optimizing the secure zone and array excitations are expected solutions.

S6.2 Comparison with other existing algorithms about scalability

We compare the proposed ADMM-based method with the genetic algorithm (GA)[6], modified Gerchberg-Saxton (MGS)[12,13], and squared-infinity norm Douglas-Rachford splitting (SQUID)[14]. Furthermore, we utilize the EVM and the convergence performance of the object value (i.e., $\|\boldsymbol{s}-\beta\mathbf{H}\boldsymbol{x}\|_2^2+K\beta^2\sigma^2$ defined in the main text) of different algorithms as the metrics. It is worth noting that to make a fair comparison, the parameters in the GA, MGS, and SQUID algorithms are configured according to the recommended values of the corresponding reference papers.

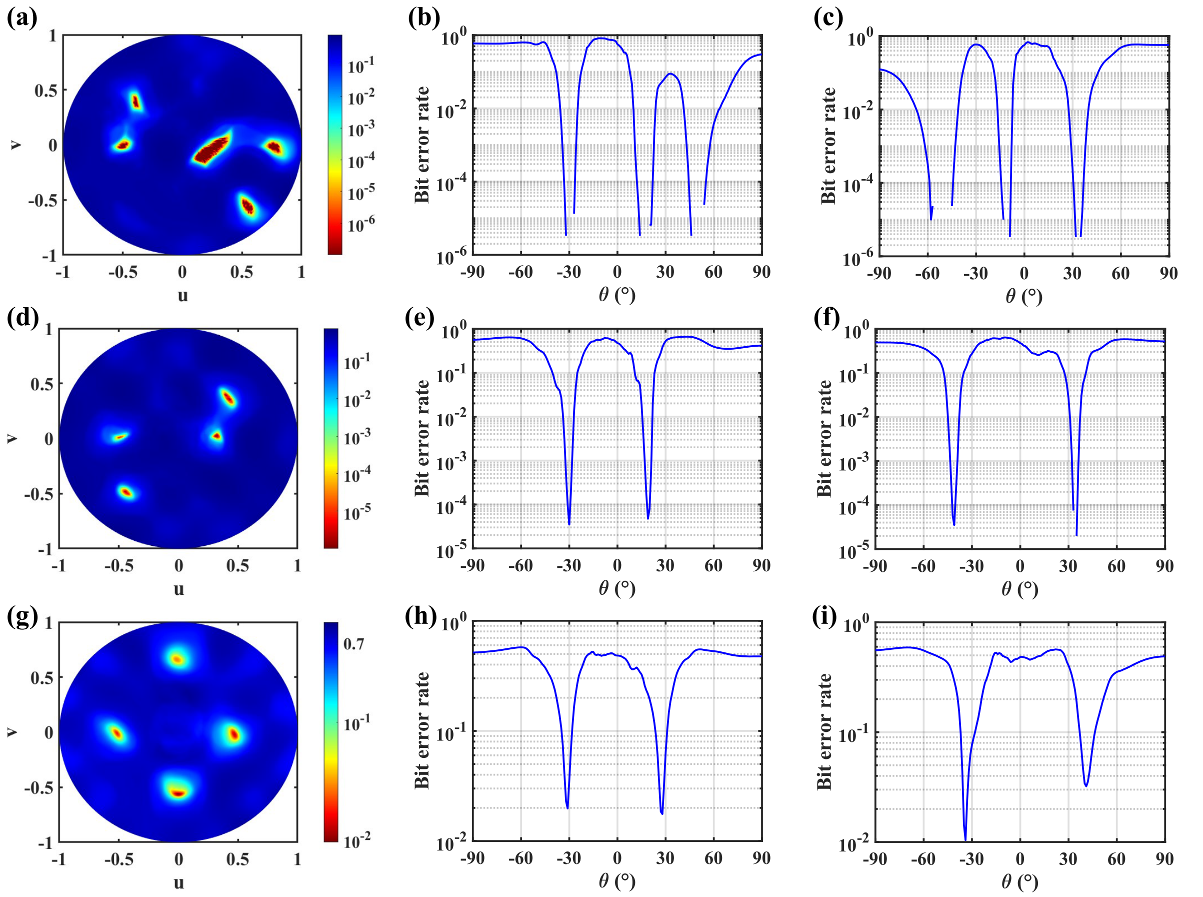

Figure S9. The BER distributions versus the elevation angle with SNR = 10 dB and 2-bit quantization. (**a-c**) Case I for 8PSK with (**a**) 2D distribution, (**b**) cut view along $\varphi = 0°$, and (**c**) cut view along $\varphi = 135°$. (**d-f**) Case II for 16QAM with (**d**) 2D distribution, (**e**) cut view along $\varphi = 0°$, and (**f**) cut view along $\varphi = 45°$. (**g-i**) Case III for 64QAM with (**g**) 2D distribution, (**h**) cut view along $\varphi = 0°$, and (**i**) cut view along $\varphi = 90°$.

We perform two representative simulation cases, as summarized in Table S3, to demonstrate the scalability of the algorithms for multiple users and different scale metasurfaces. Specially, we utilize the simulation of Case I to demonstrate the performance of the algorithms with different scale metasurface. We utilize the simulation of Case II to demonstrate the performance of the algorithms with different scale users. In Case I, there are four desired directions, in which the first two are along $\theta = 20°$ and $-30°$, respectively, on the $\varphi = 0°$ plane; while the last two are along $\theta = 34°$ and $-41°$, respectively, on the $\varphi = 45°$ plane. The simulations are conducted within three types of metasurface scale, including 4×4, 8×8, and 16×16. In Case

II, the metasurface scale is 16×16. The simulations are conducted within four and six desired directions, respectively. For each of the simulations, the modulation is 16QAM, the SNR is 15 dB, the symbols in different desired directions are selected randomly, and 2000 times of realization are performed in the Monte Carlo simulations. Each simulation is implemented using the ADMM, GA, MGS, and SQUID, respectively.

Table S3. Simulation configurations for validating the scalability of the algorithms

Scenario	Desired directions	Scale	Common settings
Case I	$(\theta_1=20°,\ \theta_2=-30°,\ \varphi_{1,2}=0°)$ $(\theta_3=34°, \theta_4=-41°,\ \varphi_{3,4}=45°)$	4×4	**Modulation:** 16QAM; **SNR:** 15 dB; **Simulations:** 2000 **Algorithms:** ADMM, GA, MGS, SQUID;
		8×8	
		16×16	
Case II	$(\theta_1=20°,\ \theta_2=-30°,\ \varphi_{1,2}=0°)$ $(\theta_3=34°, \theta_4=-41°,\ \varphi_{3,4}=45°)$	16×16	
	$(\theta_1=18°, \theta_2=50°, \theta_3=-30°,\ \varphi_{1,2,3}=0°)$ $(\theta_4=33°, \theta_5=-11°, \theta_6=-51°,\ \varphi_{4,5,6}=45^{c}$		

The results of Case I

Figures S10a-S10c display the variations of the object functions using different algorithms for the metasurface scales 4×4, 8×8, and 16×16, respectively, and there are four desired directions. As shown in Figure S10a, the GA algorithm has the best convergence accuracy in the small-scale metasurface optimization problems, while the proposed ADMM algorithm has the worst performance. We should admit that the proposed algorithm is not competitive when dealing with small-scale problems. However, when the metasurface scale increases to 8×8, the ADMM algorithm has comparable performance the benchmark SQUID, as shown in Figure S10b. Furthermore, we notice that the GA and MGS algorithms quickly fall into the local minimum value. Figure S10c demonstrates the case of higher scale 16×16, where our algorithm has the best convergence rate and accuracy, while the SQUID is unable to converge.

Below each corresponding convergence picture, we plot the EVM distribution as the functions of the elevation angle in the $\varphi=0°$ plane and in the $\varphi=45°$ plane, respectively. As shown

in Figure S10d, the lowest EVM values are located right at the desired directions, while the values in other directions are relatively larger, thus validating the directional secure property of the system. Comparing the results shown in Figures S10d-S10f, we observe that with the increase of metasurface scale, the EVM values optimized by the ADMM algorithm decreases continuously in the desired directions, indicating that our proposed method can handle the large-scale problems and has better performance.

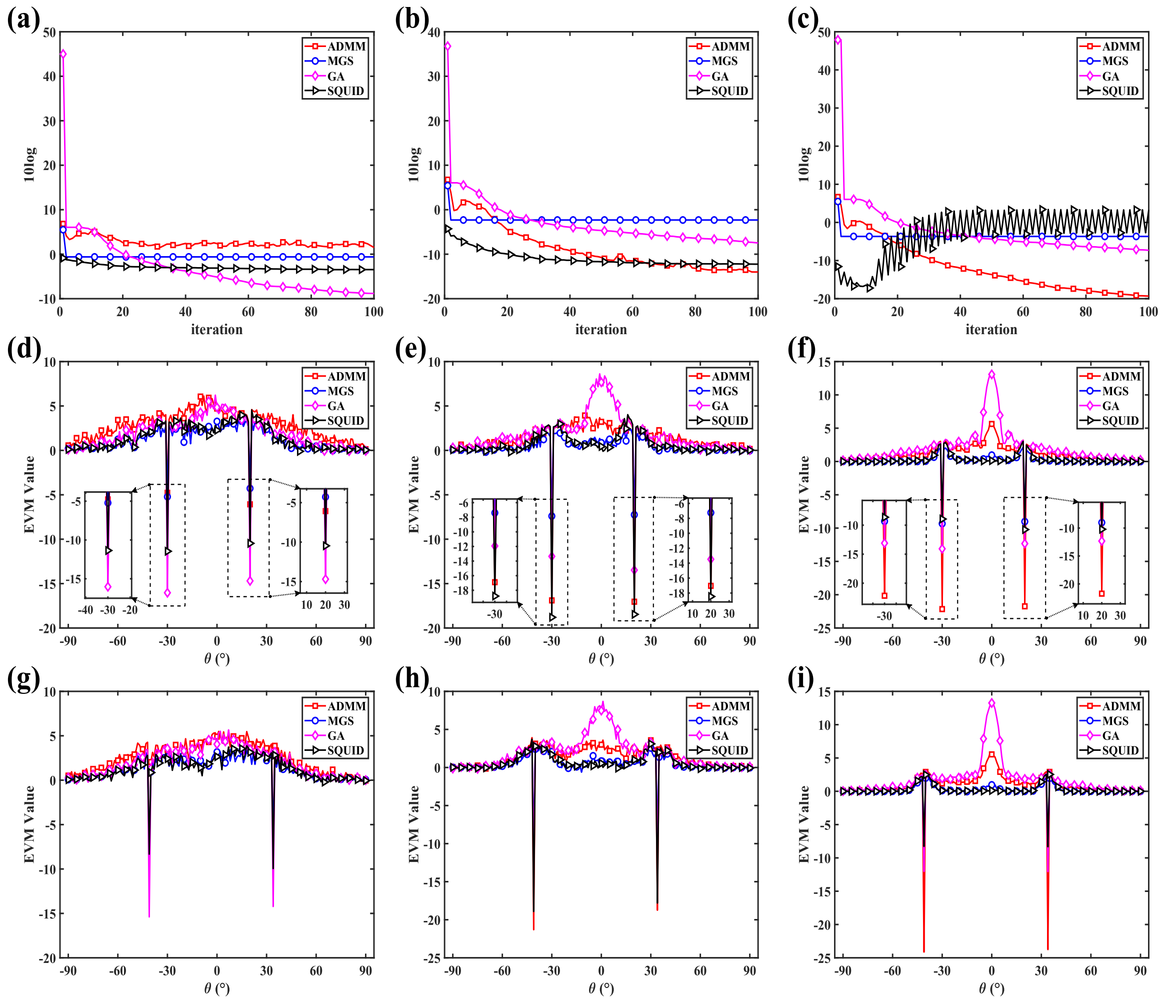

Figure S10. Simulations that validate the performance of the algorithms with different metasurface scales. There are four desired directions, including $(\theta_1, \varphi_1) = (20°, 0°)$, $(\theta_2, \varphi_2) = (-30°, 0°)$, $(\theta_3, \varphi_3) = (34°, 45°)$, and $(\theta_4, \varphi_4) = (-41°, 45°)$ **(a-c)** Variations of the object functions for three metasurface scales, 4×4, 8×8, and 16×16, respectively. (**d**), (**g**) The EVM distributions as the function of the elevation angles in the $\varphi = 0°$ plane and $\varphi = 45°$ plane, respectively, and the metasurface scale is 4×4. (**e**), (**h**) The EVM distributions as the function of the elevation angles in the $\varphi = 0°$ plane and $\varphi = 45°$ plane, respectively, and the metasurface scale is 8×8. (**f**), (**i**) The EVM distributions as the

function of the elevation angles in the $\varphi=0^\circ$ plane and $\varphi=45^\circ$ plane, respectively, and the metasurface scale is 16×16.

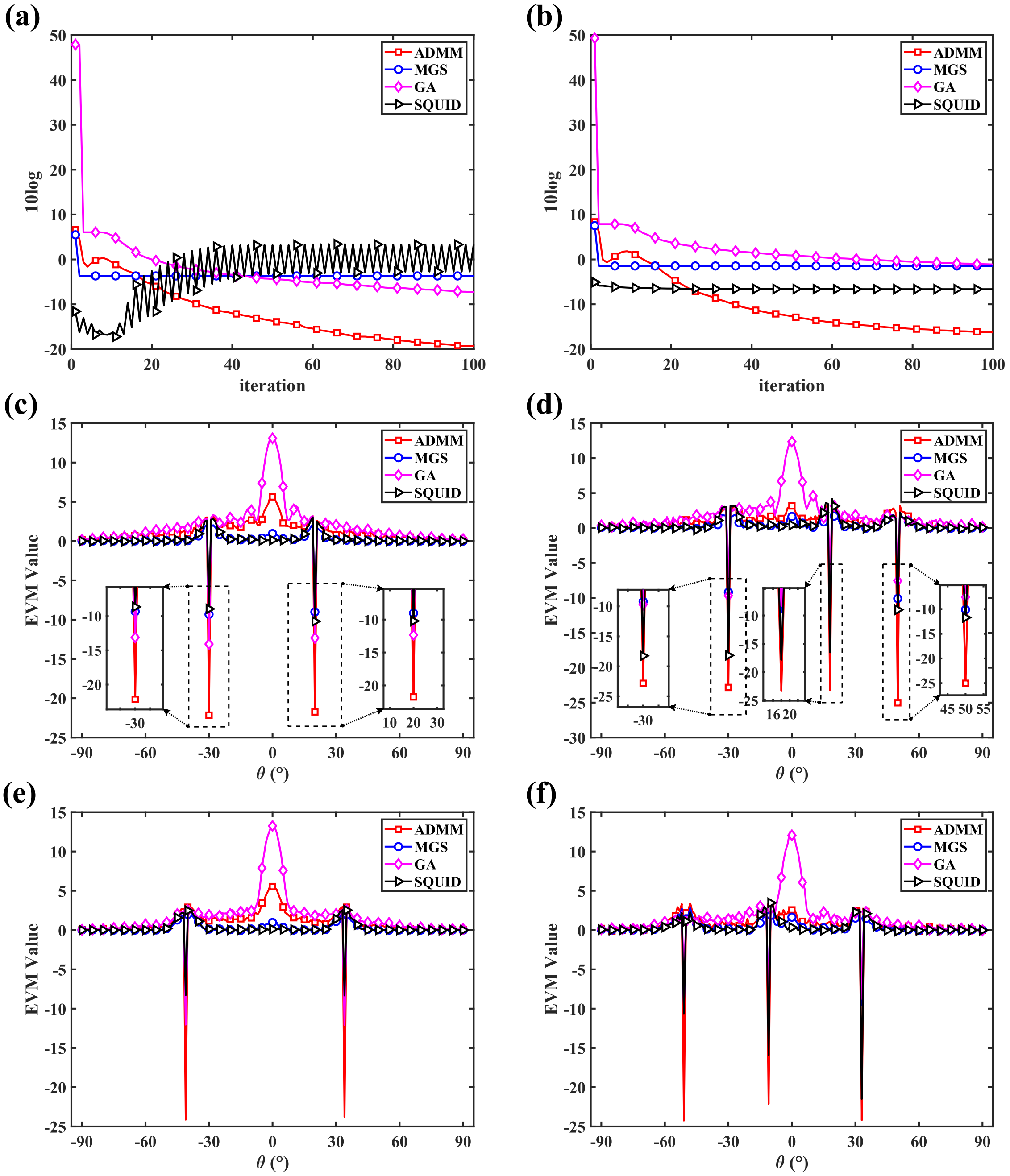

Figure S11. Simulations that validate the performance of the algorithms with different number of users. The metasurface scale is 16×16. (**a**), (**b**) Variations of the object functions when the number of users are four and six desired directions, respectively. (**c**), (**d**) The EVM distributions as the function of the elevation angles in the $\varphi=0^\circ$ and $\varphi=45^\circ$ planes, respectively, and there are four users. (**e**), (**f**) The EVM distributions as the function of the elevation angles in the $\varphi=0^\circ$ plane and $\varphi=45^\circ$ plane, respectively, and there are six users.

The results of Case II

Figures S11a and S11b demonstrate the variations of the object functions using different algorithms for four and six desired directions, respectively, in which the metasurface scale is 16×16. The results about the four-channel case have been analyzed in the previous section. Our attention turns to the results of the six desired directions. As shown in Figure S11b, the proposed ADMM method has the best convergence performance compared with other algorithm, and the GA, MGS, SQUID quickly fall into the local minimum values. The results demonstrate that our method can solve larger-scale problems. Furthermore, the lowest EVM shown in Figures S11c and S11d are in the desired directions. The EVM values optimized by the ADMM are lowest, which matches well with the convergence results.

Note 7. Process of the measuring signals

As mentioned in the main text, we assess the quality of the received signals by comparing them with the reference constellation diagram. We conduct three steps to remove the effects of propagation loss and phase shift of the received signals, including the power normalization, multiplication by β, and the addition of an identical phase bias. The power normalization ensures that the total power of the received signals is *M*, where *M* is the number of the corresponding constellation symbols. This process guarantees that the magnitudes of the received signals are comparable with the reference constellation symbols. The process formula is

$$\mathbf{s}_1 = \frac{\mathbf{s}_m}{\sqrt{\frac{1}{M}\sum_{i=1}^{M} |s_{m,i}|^2}}, \tag{S16}$$

where $\mathbf{s}_1$ and $\mathbf{s}_m$ are the normalized signals and the received signals, respectively.

The optimized β factor provides the additional magnitude information that helps to reduce the difference between the received and reference signals. In this study, we assume that users know the β factor well. After multiplication by β, the signals are $s_{2,i} = \beta_i s_{1,i}, \quad i = 1, 2, ..., M$.

The addition of an identical phase bias removes the impact of the propagation phase shift. To

find the optimal phase bias, we formulate a univariate and unconstrained optimization, namely,

$$\gamma^* = \arg\min_{\gamma} \left\| \mathbf{s} - e^{j\gamma}\mathbf{s}_2 \right\|_2^2, \tag{S17}$$

where γ^* and s are the optimized phase bias and the reference constellation symbols, respectively. The closed-form solution to the problem is $\gamma^* = \frac{j}{2}\ln \mathbf{s}_2^H \mathbf{s}\mathbf{s}_2^H \mathbf{s} \Big/ \left\| \mathbf{s}_2^H \mathbf{s} \right\|_2^2$.

As an illustrated example, the process for measuring the 8PSK signals is shown in Figure S12. After the power normalization, the magnitudes of the received signals are comparable to the reference signals. The β factor further adjusts the magnitude of the received signals. The addition of the optimized phase bias ensures a perfect agreement between the received signals and the reference symbols.

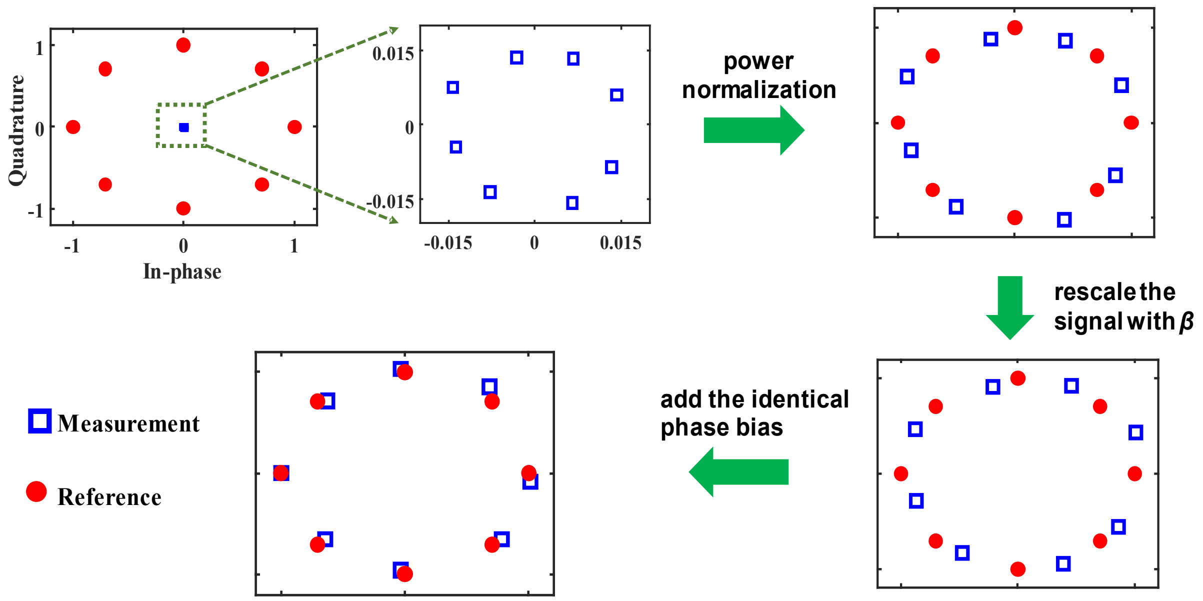

Figure S12. The process for measuring the 8PSK signals.

Note 8. Measurements of the four-channel modulations

We conducted new experiments with up to four simultaneous users, whose locations are $(\theta, \varphi) = (12°, 0°)$, $(\theta, \varphi) = (-30°, 0°)$, $(\theta, \varphi) = (42°, 90°)$, and $(\theta, \varphi) = (-34°, 90°)$, respectively. The performasnce of DIM for more users is verified using the 8PSK, 16QAM, and 64QAM schemes, respectively. We here briefly summarize the data and figures for each scheme.

For the 8PSK scheme, we present the magnitudes and phases of measured fields, the received constellation diagrams of each user, the optimized coding sequences of the 8×8 programmable metasurface, and the EVM distribution over the elevation angles for validating the directional security. For the 16QAM scheme, apart from the above data, we also perform the cross-talk measurements and draw the corresponding matrix values. For the 64QAM scheme, we only present the received constellation diagrams of each user and the EVM distribution for the consideration of simplicity and clarity.

8.1 The results of four-channel 8PSK scheme

Figures S13a and S13b show the magnitudes and phases of the measured fields for the 8PSK scheme along the $\varphi=0°$ plane. In this case, the main lobe beams are located near the target directions $\theta=12°$ and $\theta=-30°$, respectively, and the radiation powers are almost equal for all symbols. Another interesting observation is that the phases of measured fields have an interval of nearly 45° in the vicinity of the target directions. Figures S13c and S13d show the results along the $\varphi=90°$ plane, where we observe the similar features in the target directions $\theta=42°$ and $\theta=-34°$. The above results demonstrate that the simultaneous four-channel 8PSK modulation is realized with the help of our DIM system. More intuitionistic evidence is shown in Figure S14, in which the measured symbols are in excellent agreement with the references. The optimized digital coding distributions of the 8×8 PM that implements the four-channel 8PSK modulation are demonstrated in Figure S15.

The EVM distribution as the functions of the elevation angles for the 8PSK scheme is demonstrated in Figure S16. The EVM values in the vicinity of the target directions are relatively lower, which validates the security property of the proposed method. We should explain the observation that the EVM values are low near the direction $\theta=0°$ along the $\varphi=90°$ plane shown in Figure S16b. The reason behind this is that the measured fields in this part are also within the secure zone of user 1 (i.e., in the vicinity of the direction $(\theta,\ \varphi)=(12°,\ 0°)$). We also present the measured constellation in the unintended directions

shown in the bottom subfigures, in which the signal structures are distorted.

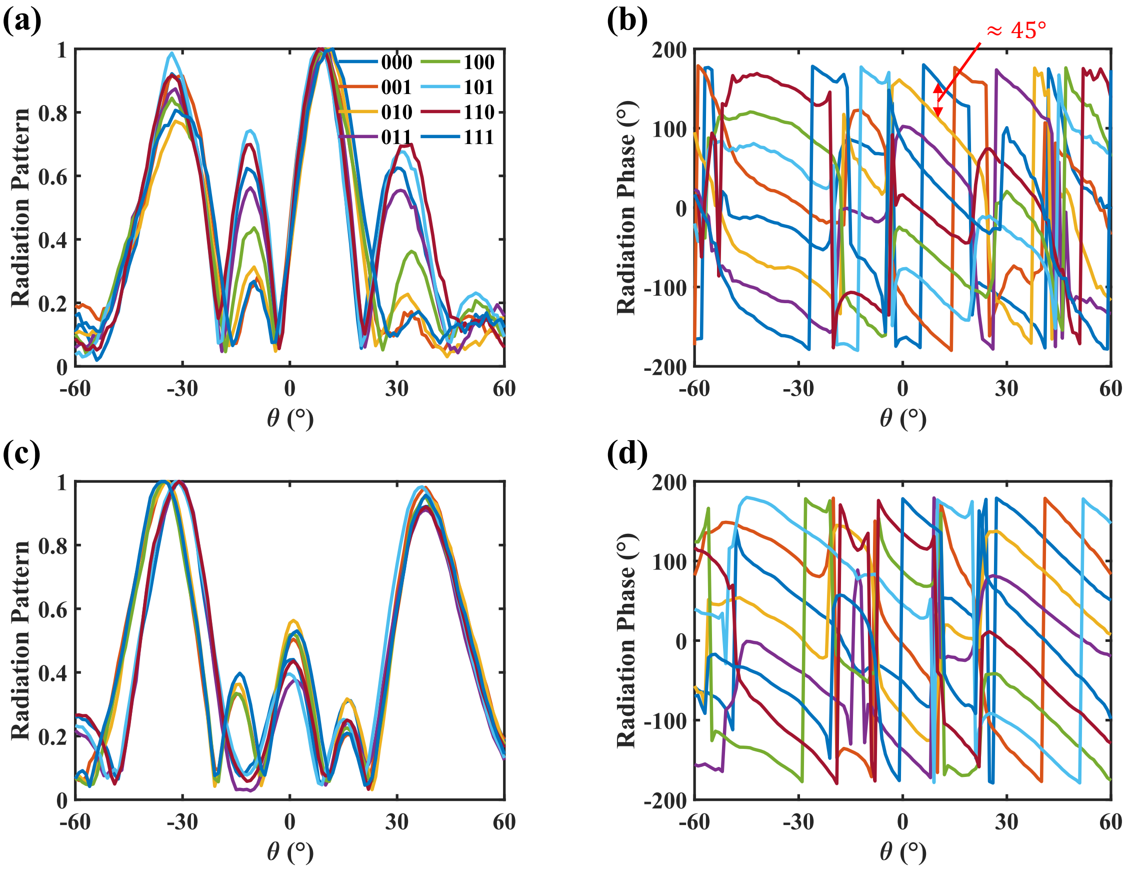

Figure S13. The measured fields in the four-channel 8PSK experiment. (**a**)-(**b**) The results in the $\varphi = 0°$ plane. (**c**)-(**d**) The results in the $\varphi = 90°$ plane.

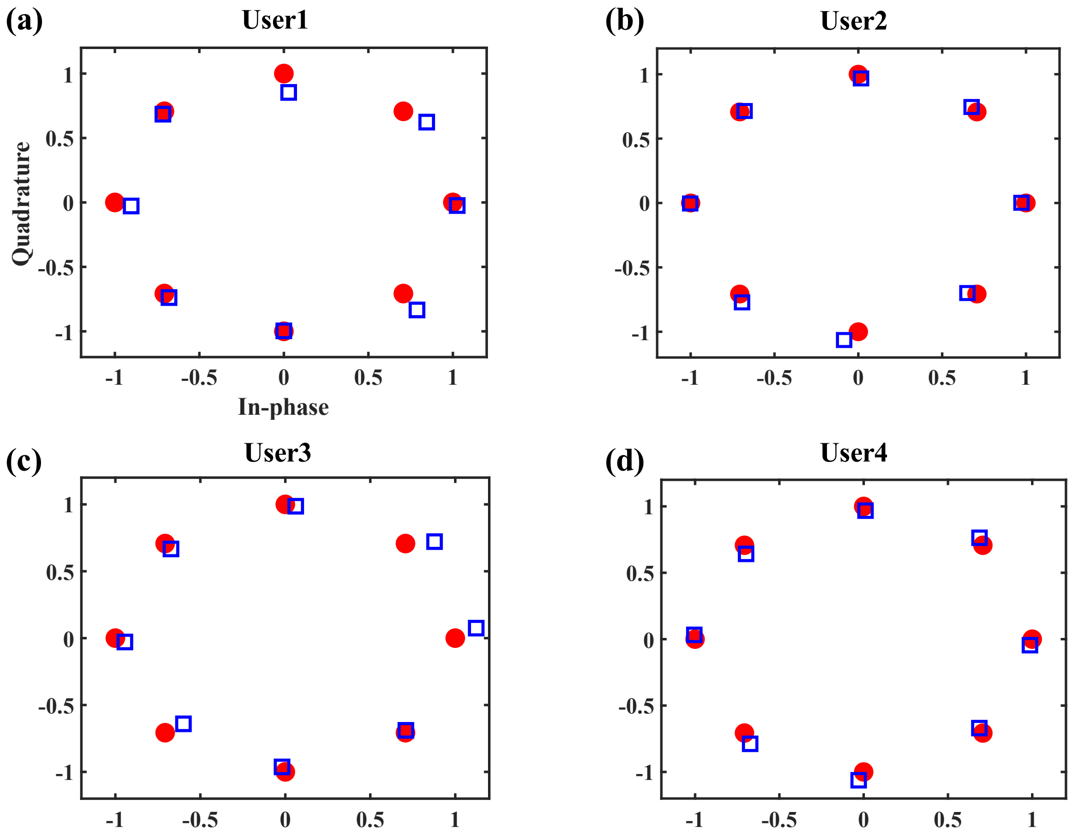

Figure S14. The measured constellation diagrams of the four-channel 8PSK experiment. The measured and the reference symbols are represented by the blue square and the red circular markers, respectively. (**a**) The diagrams of user 1 located at $(\theta, \varphi) = (12°, 0°)$. (**b**) The diagrams of user 2 located at $(\theta, \varphi) = (-30°, 0°)$. (**c**) The diagrams of user 3 located at $(\theta, \varphi) = (42°, 90°)$. (**d**) The diagrams of user 4 located at $(\theta, \varphi) = (-34°, 90°)$.

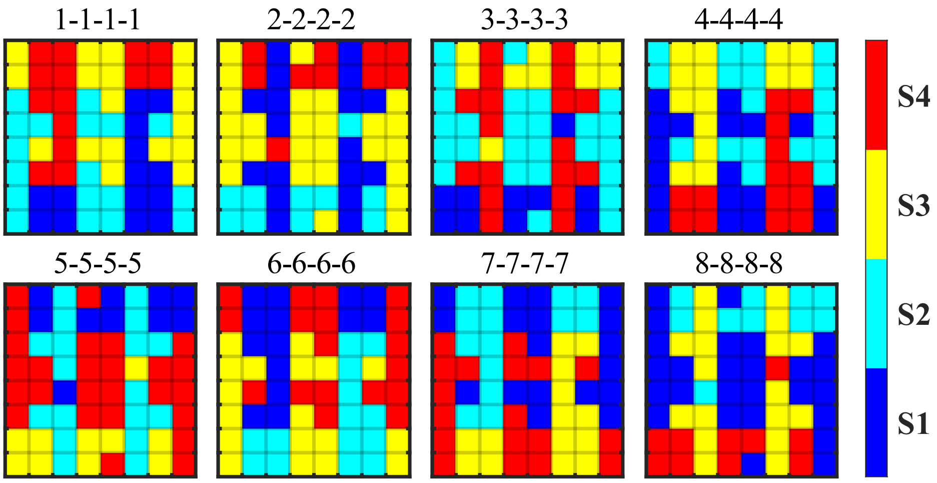

Figure S15. The optimized coding distributions of the 8×8 programmable metasurface that implement four-channel 8PSK. (S1: $-90°$, S2: $0°$, S3: $90°$, S4: $180°$)

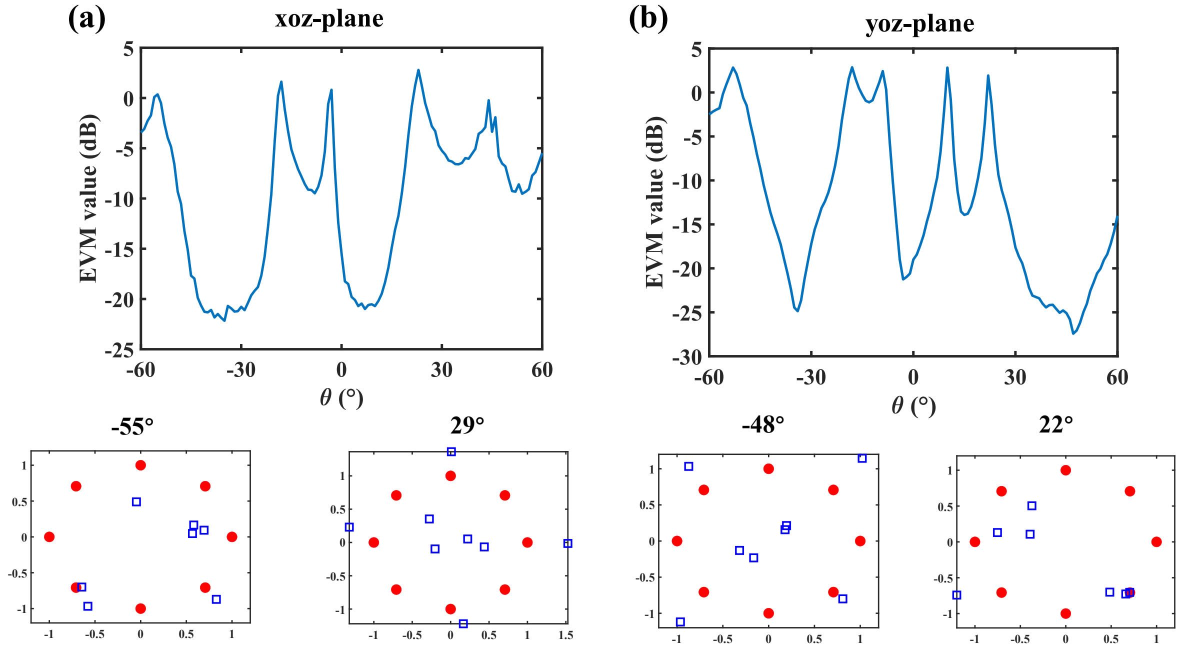

Figure S16. The measured EVM values in the four-channel 8PSK experiment. (**a**) The EVM distributions in the $\varphi = 0°$ plane. The bottom subfigures present two constellation diagrams that deviate from the desired users. (**b**) The EVM distributions along the $\varphi = 90°$ plane. The bottom subfigures present two constellation diagrams that deviate from the desired users.

8.2 The results of four-channel 16QAM scheme

Figures S17a and S17b present the magnitudes and phases of the measured fields along the $\varphi = 0°$ plane associated with the 16QAM, respectively. As illustrated in Figure S17a, the radiation patterns form three clusters near the desired directions $\theta = 12°$ and $\theta = -30°$, and the average powers of each cluster are presented. The radiation phases of measured fields are regularly arranged in the vicinity of the target directions. Figures S17c and S17d show the results along the $\varphi = 90°$ plane, where we observe the similar features in the target directions $\theta = 42°$ and $\theta = -34°$. The above results demonstrate that the simultaneous four-channel 16QAM is realized. More intuitionistic evidence is shown in Figure S18, in which the measured symbols are in good agreement with the reference.

The EVM distribution as the functions of the elevation angles is demonstrated in Figure S19. The EVM values in the vicinity of the target directions are relatively lower than in other

directions, which validates the security property of the proposed method.

For a multi-user communication system, the evaluation of cross-talk value is crucial because it determines whether the channels are independent. However, to the best of authors' knowledge, the definition and calculation method of multi-user crosstalk in the field of DIM are still absent. For filling up this gap, we try to quantify the crosstalk for multiple users in terms of a matrix **C** in which the matrix is composed of $K \times K$ elements c_{ij} (K is the number of users). We should first explain the motivations for calculating cross-talk values. In the field of electrical or optical communications, the crosstalk is defined as the cross-power coupling between multiple channels[15]. When the input signal of one channel changes, receivers measure the variation of signals at the other channels. According to the difference values, the magnitudes of crosstalk are obtained. We here mimic the operation and perform the corresponding the experiments. For calculating the element $c_{ij}\,(i \neq j, j = 1, 2, ..., i-1, i+1, ..., K)$, we perform the experiment that the transmitter simultaneously sends M different symbols to user i and the M same symbols to the other users. The c_{ij} $(i \neq j)$ is

$$c_{ij} = \sqrt{\sum\nolimits_{m=1}^{M} |\,\boldsymbol{s}_{mea}^{j}(m) - \boldsymbol{s}_{ref}^{j}(m)\,|^2 \Big/ \sum\nolimits_{m=1}^{M} |\,\boldsymbol{s}_{ref}^{j}(m)\,|^2}\,, \tag{S18}$$

where $\boldsymbol{s}_{mea}^{j}$ and $\boldsymbol{s}_{ref}^{j}$ are the measured and reference symbols of user j. Finally, the value of c_{ii} is $\sqrt{1 - \sum\limits_{j \neq i} c_{ij}^2}$.

To measure the crosstalk values of four users using the proposed method, we conducted four independent experiments. In the first group of experiments, the sixteen different symbols are sent to user 1, and the same symbols coded as "0011" are sent to user 2, and the same symbols coded as "1001" are sent to user 3, and the same symbols coded as "1100" are sent to user 4. In the second group of experiments, the sixteen different symbols are sent to user 2, and the same symbols coded as "1011" are sent to user 1, and the same symbols coded as "0110" are sent to user 3, and the same symbols coded as "1110" are sent to user 4. In the third group of experiments, the sixteen different symbols are sent to user 3, and the same symbols coded as "1100" are sent to user 1, and the same symbols coded as "0011" are sent to user 2, and the

same symbols coded as “1110” are sent to user 4. In the fourth group of experiments, the sixteen different symbols are sent to user 4, and the same symbols coded as “1011” are sent to user 1, and the same symbols coded as “0001” are sent to user 2, and the same symbols coded as “0011” are sent to user 3.

The measurements are presented in Figures S20a-S20d. Taking Figure S20a as an illustrative example, we observe that the symbols received by user 1 are in good agreement with the 16QAM, while the signals of user 2, 3, and 4 are clustered near the corresponding reference point. The results demonstrate that our system has good crosstalk resistance. We then calculate the whole cross-talk matrix **C** according to the Equation (S18). As show in Figure S21, the cross-talk values between difference users are relatively low and the maximum is 0.24 (-12.4 dB), implying the acceptable performance under the four-channel transmission.

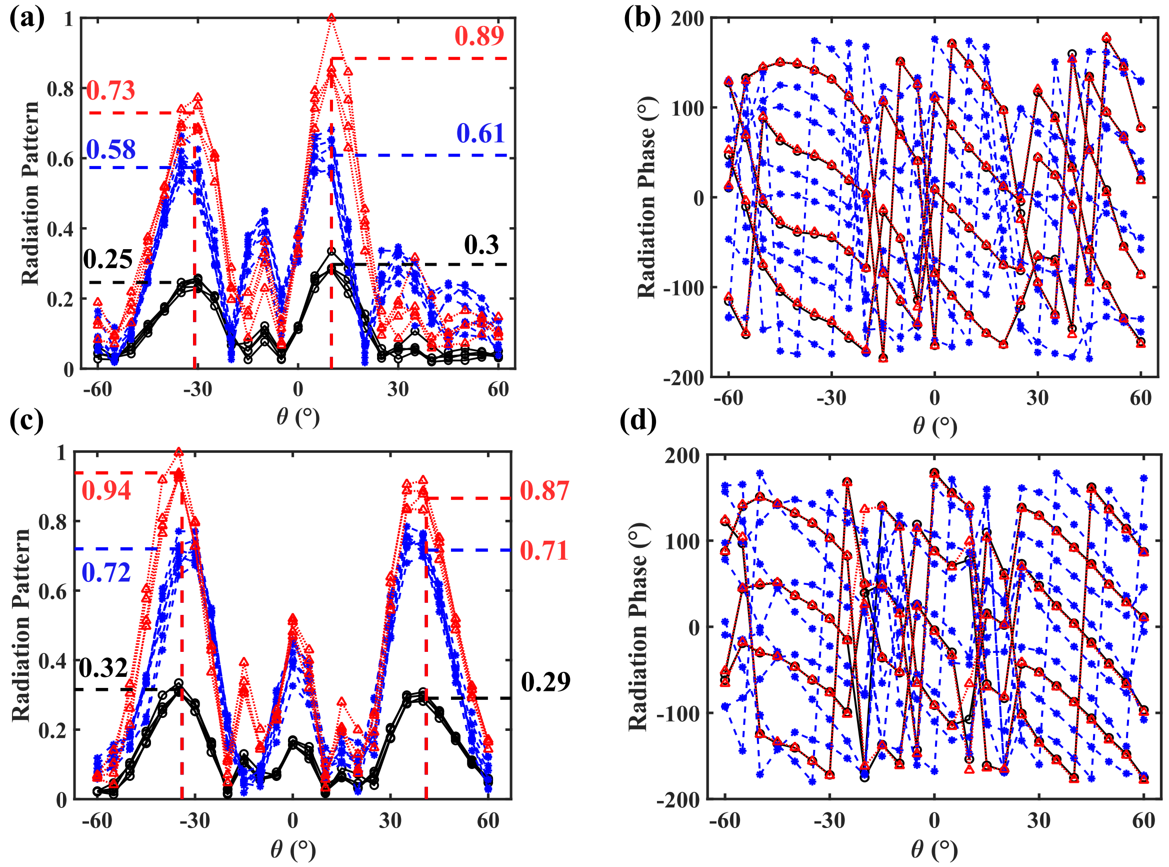

Figure S17. The measured fields in the four-channel 16QAM scheme. (**a**)-(**b**) The results in the $\varphi = 0°$ plane. (**c**)-(**d**) The results in the $\varphi = 90°$ plane.

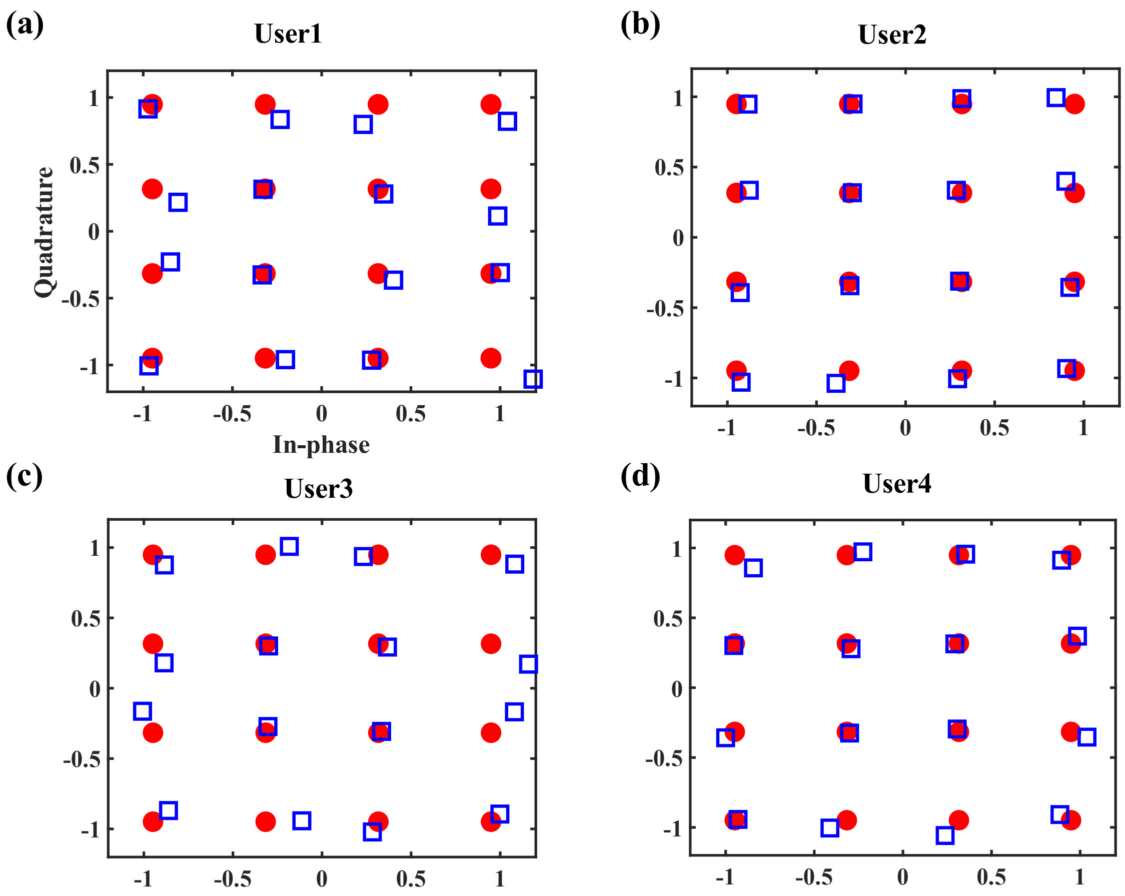

Figure S18. The measured constellation diagrams of the four-channel 16QAM experiment. The measured and the reference symbols are represented by the blue square and the red circular markers, respectively. (**a**) The diagrams of user 1 located at $(\theta,\ \varphi) = (12°,\ 0°)$. (**b**) The diagrams of user 2 located at $(\theta,\ \varphi) = (-30°,\ 0°)$. (**c**) The diagrams of user 3 located at $(\theta,\ \varphi) = (42°,\ 90°)$. (**d**) The diagrams of user 4 located at $(\theta,\ \varphi) = (-34°,\ 90°)$.

$$\mathbf{C} = \begin{bmatrix} c_{11} & c_{12} & c_{13} & c_{14} \\ c_{21} & c_{22} & c_{23} & c_{24} \\ c_{31} & c_{32} & c_{33} & c_{34} \\ c_{41} & c_{42} & c_{43} & c_{44} \end{bmatrix} = \begin{bmatrix} 0.95 & 0.13 & 0.23 & 0.13 \\ 0.15 & 0.97 & 0.13 & 0.13 \\ 0.23 & 0.18 & 0.95 & 0.13 \\ 0.24 & 0.17 & 0.19 & 0.94 \end{bmatrix}$$

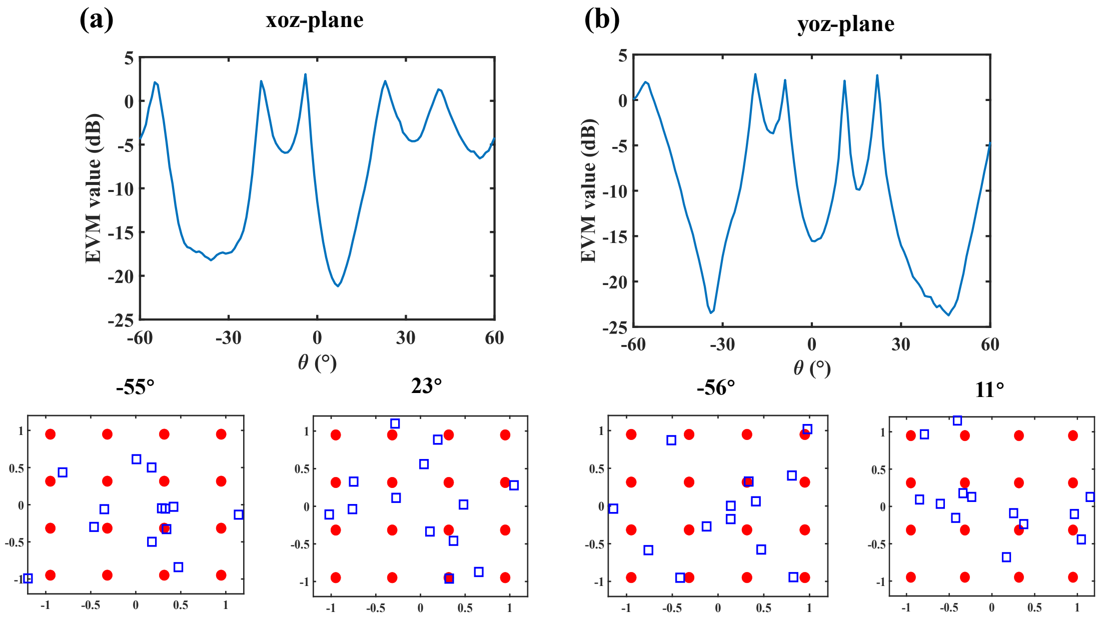

Figure S19. The measured EVM values in the four-channel 16QAM experiment. (**a**) The EVM distribution in the $\varphi = 0°$ plane. The bottom subfigures present two constellation diagrams that deviate from the desired users. (**b**) The EVM distribution in the $\varphi = 90°$ plane. The bottom subfigures present two constellation diagrams that deviate from the desired users.

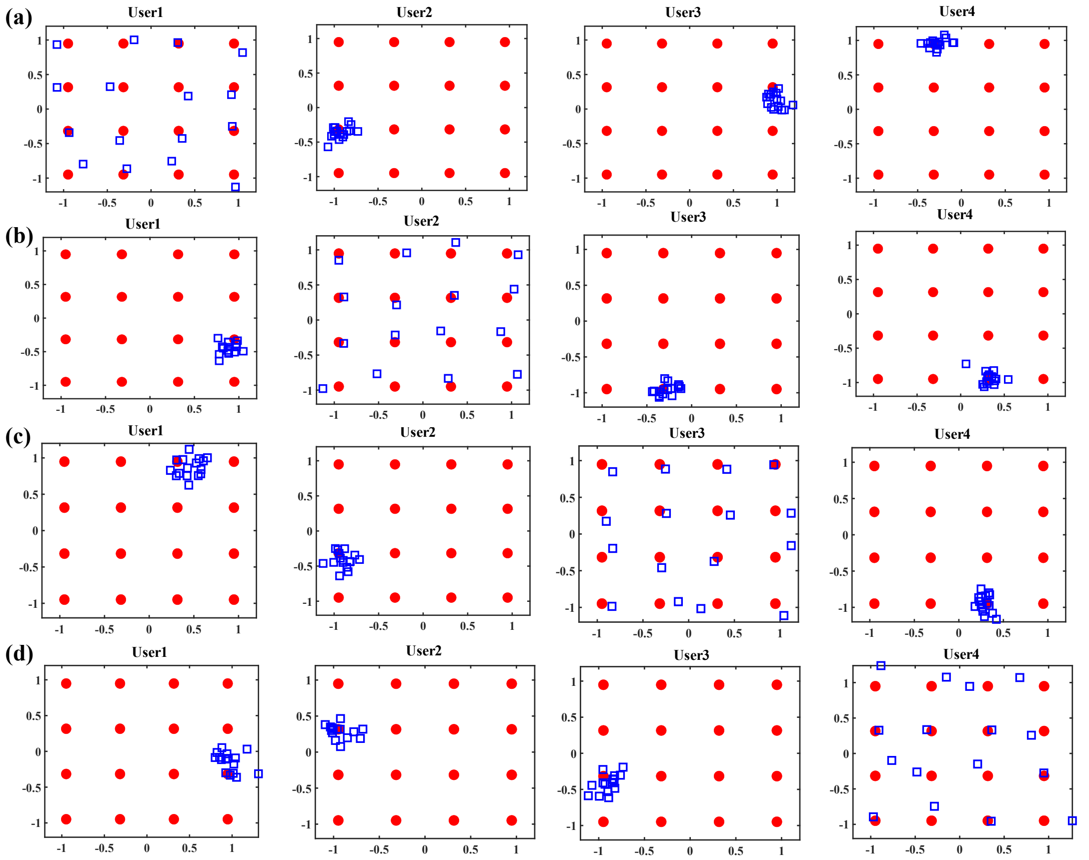

Figure S20. The measured constellation diagrams of each user in the experiments that measure the cross-talk

values of multiple users. The measured and the reference symbols are represented by the blue square and the red circular markers, respectively. (**a**) The diagrams of the first group of experiments. (**b**) The diagrams of the second group of experiments. (**c**) The diagrams of the third group of experiments. (**d**) The diagrams of the fourth group of experiments.

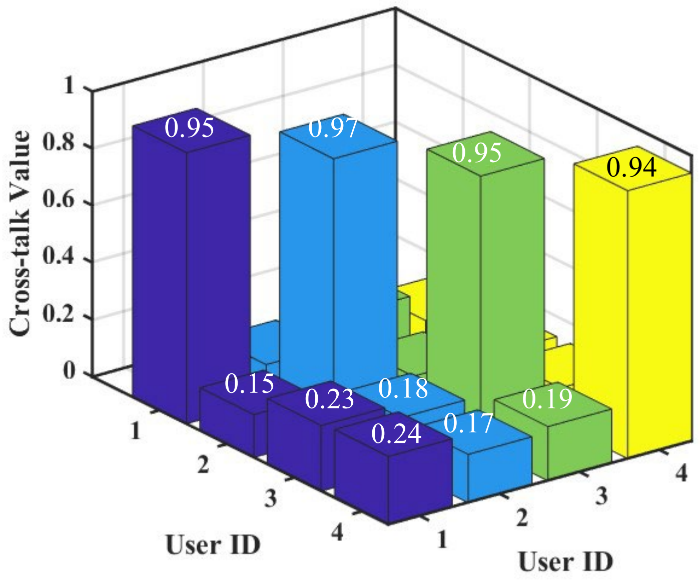

Figure S21. The cross-talk values of four users.

8.3 The results of four-channel 64QAM scheme

We present the measurements of four-channel 64QAM scheme. Such configuration is more difficult than the previous experiments since the modulation has higher order, and the distances between adjacent symbols are closer. Figure S22 shows the measured symbols of each user, in which the results of user 3 and 4 are relatively better than user 1 and 2. The EVM distribution as the functions of the elevation angles is given in Figure S23. The EVM values in the vicinity of the target directions are relatively lower than in other directions, which validates the security property of the proposed method.

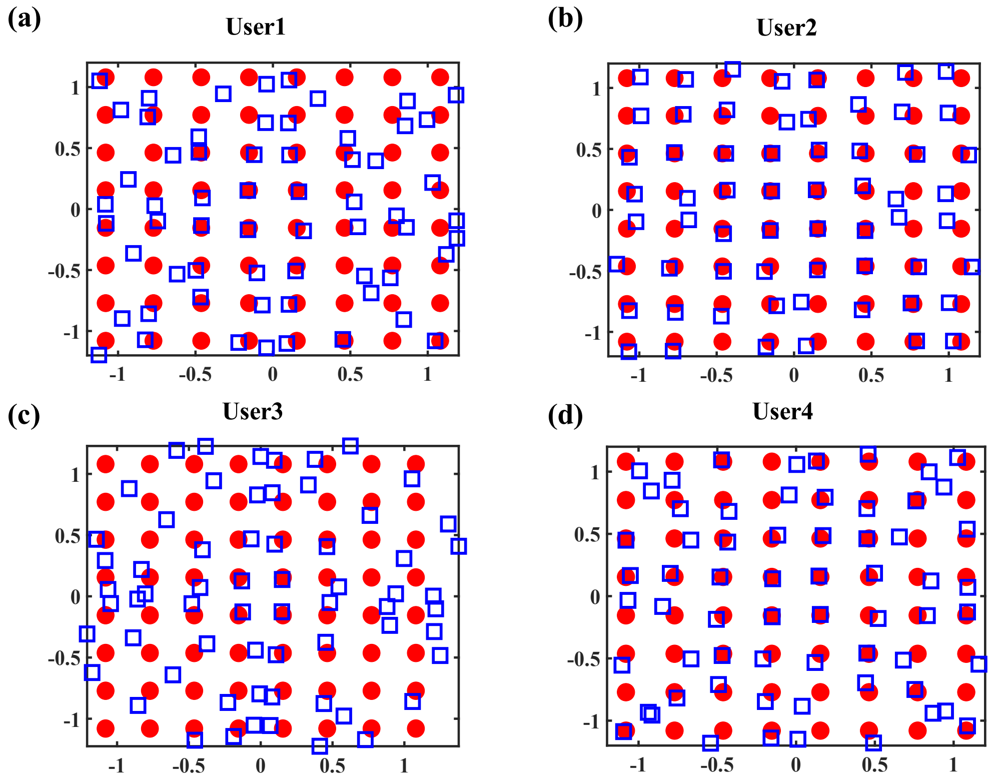

Figure S22. The received constellation diagrams in the four-channel 64QAM experiment. The measured and the reference symbols are represented by the blue square and the red circular markers, respectively. (**a**) The diagrams of user 1 located at $(\theta,\ \varphi)=(12°,\ 0°)$. (**b**) The diagrams of user 2 located at $(\theta,\ \varphi)=(-30°,\ 0°)$. (**c**) The diagrams of user 3 located at $(\theta,\ \varphi)=(42°,\ 90°)$. (**d**) The diagrams of user 4 located at $(\theta,\ \varphi)=(-34°,\ 90°)$.

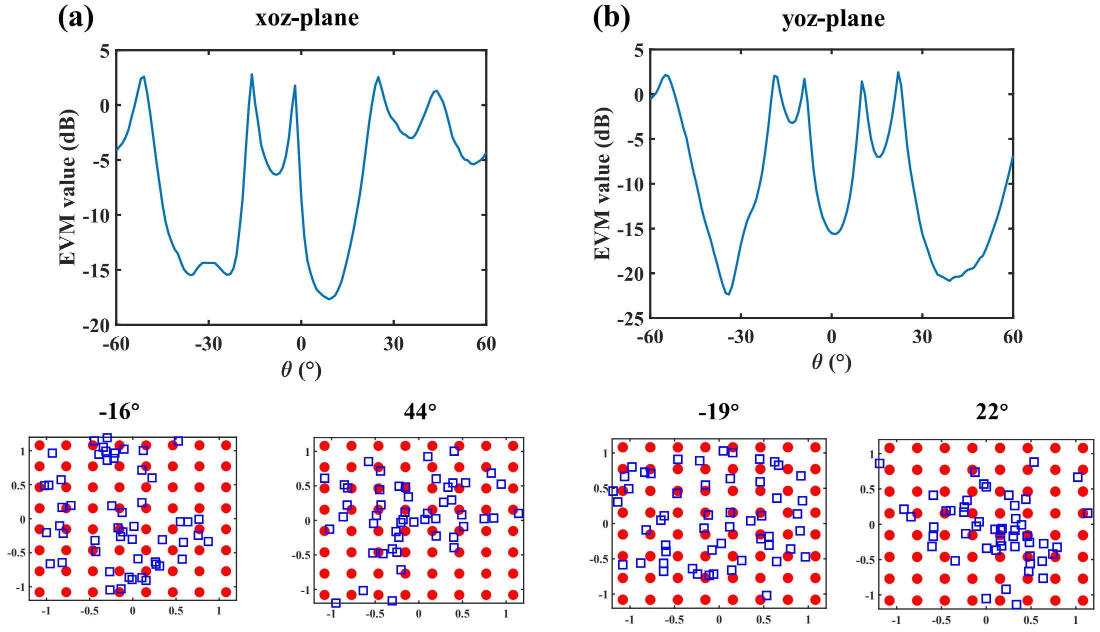

Figure S23. The EVM values as the functions of the elevation angles in the four-channel 64QAM scheme.

(**a**) The EVM distribution in the $\varphi = 0°$ plane. The bottom subfigures present two constellation diagrams that deviate from the desired users. (**b**) The EVM distribution in the $\varphi = 90°$ plane. The bottom subfigures present two constellation diagrams that deviate from the desired users.

Note 9. The magnitudes and phases of the measured fields for experiments

We added the data and figures of the measured fields for the two experiments in the main text. The first experiment is the single-channel 8PSK and 64QAM. The second experiment is the cross-talk measurement for the dual-channel 16QAM.

9.1 The measured fields for the single-channel 8PSK and 64QAM

Figures S24a and S24b demonstrate the magnitudes and phases of the measured fields for the single-channel 8PSK scheme, respectively, in which the main lobe beams are along the target direction $\theta = -19°$ and the radiation powers are larger than 0.8 for all symbols. Another interesting observation is that the phases of the measured fields are distributed with nearly 45° interval in the vicinity of the target direction. Figures S24c and S24d show the magnitudes and phases of simulated fields, respectively, which demonstrate similar features to the measurements.

The magnitudes and phases of measured fields for the single-channel 64QAM scheme are shown in Figures S25a and S25b. The curves are quite dense, but we can still observe that the main lobe beams with different magnitudes are generated and radiation phases are regularly arranged in the vicinity of the target direction $\theta = 21.5°$, which matches with the characteristics of 64QAM constellation symbols.

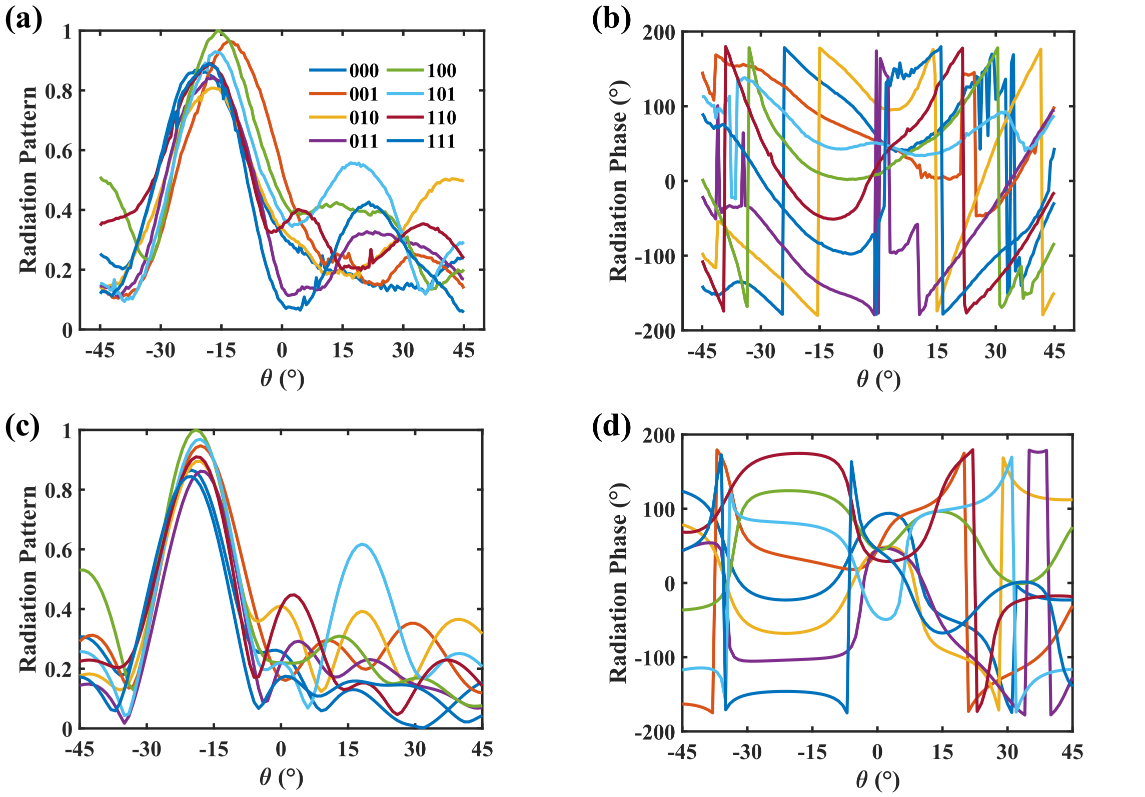

Figure S24. The fields in the single-channel 8PSK experiment. The measured (**a**) magnitudes and (**b**) phases of fields. The simulated (**c**) magnitudes and (**d**) phases of fields.

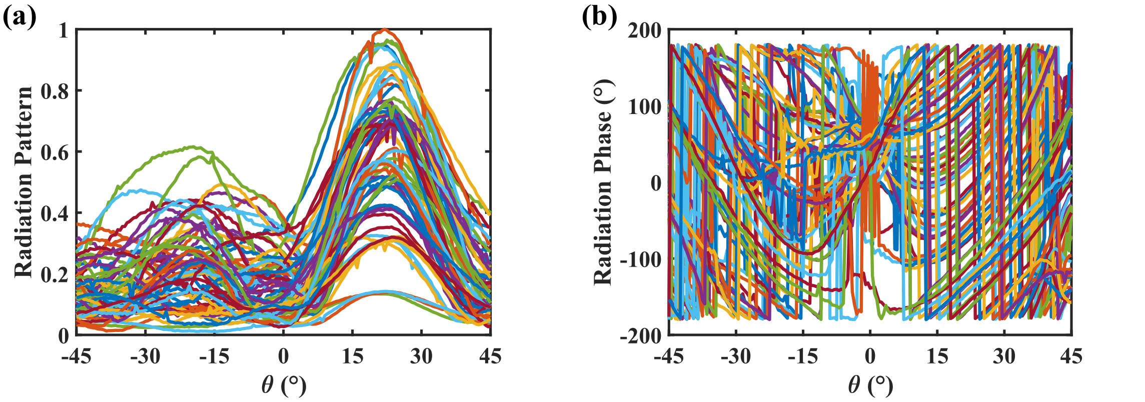

Figure S25. The fields in the single-channel 64QAM experiment. The measured (**a**) magnitudes and (**b**) phases of fields.

9.2 The measured fields for the cross-talk experiment

In order to obtain the complete crosstalk matrix, we have conducted two independent experiments. Specifically, In the first group of experiments, the sixteen different symbols are sent to user 1, and the same symbols coded as “0000” are sent to user 2. In the second group of

experiments, the sixteen different symbols are sent to user 2, and the same symbols coded as "0011" are sent to user 2. The results are shown in Figures S26 and S27, which matches well with the transmitted symbols.

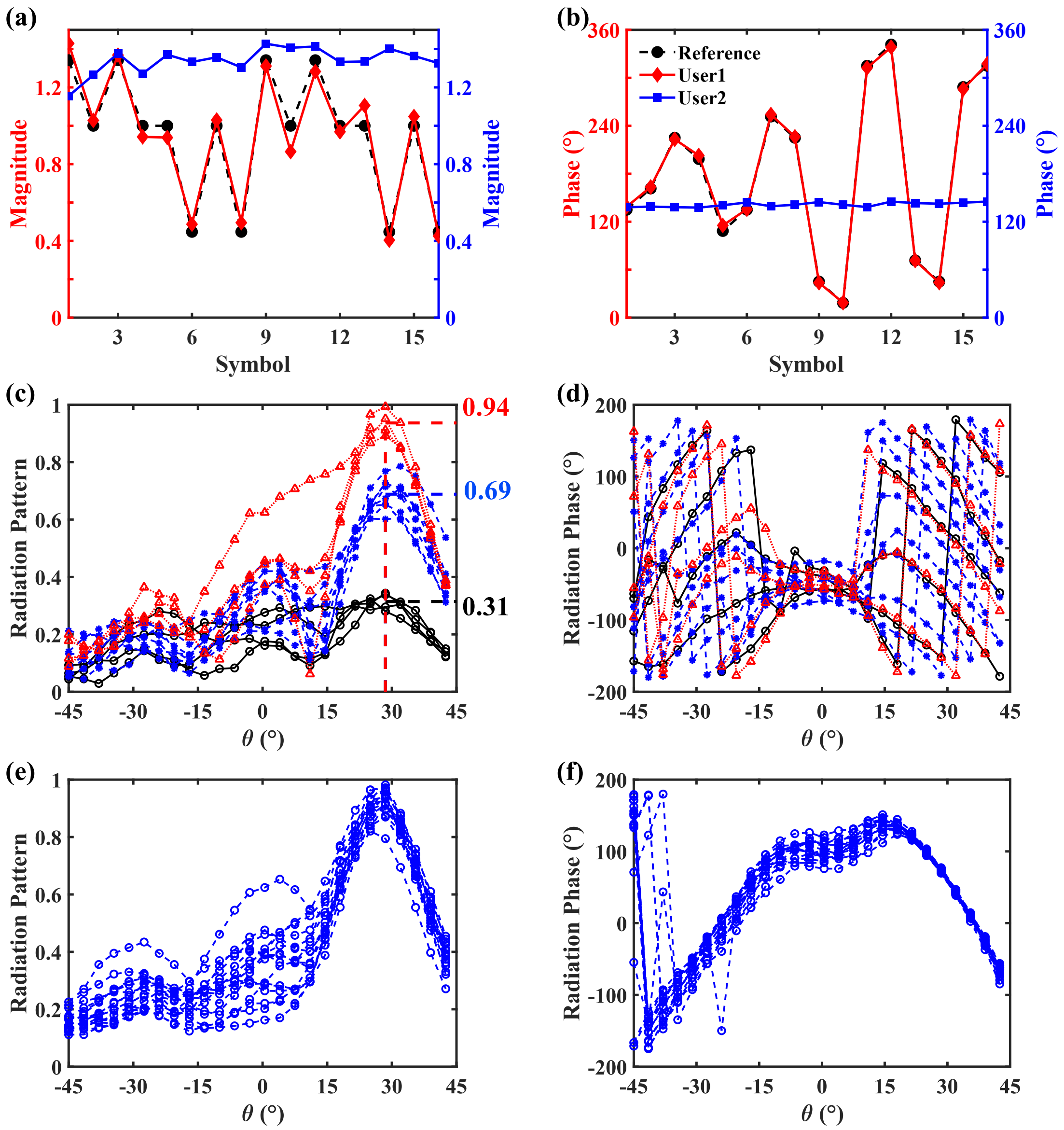

Figure S26. Results of the first group of tests for the dual-channel 16QAM. (**a**) The magnitudes and (**b**) phases of the signals in the desired directions. (**c**) The magnitudes and (**d**) phases of fields in the $\varphi = 0°$ plane. (**e**) The magnitudes and (**f**) phases of fields in the $\varphi = 90°$ plane.

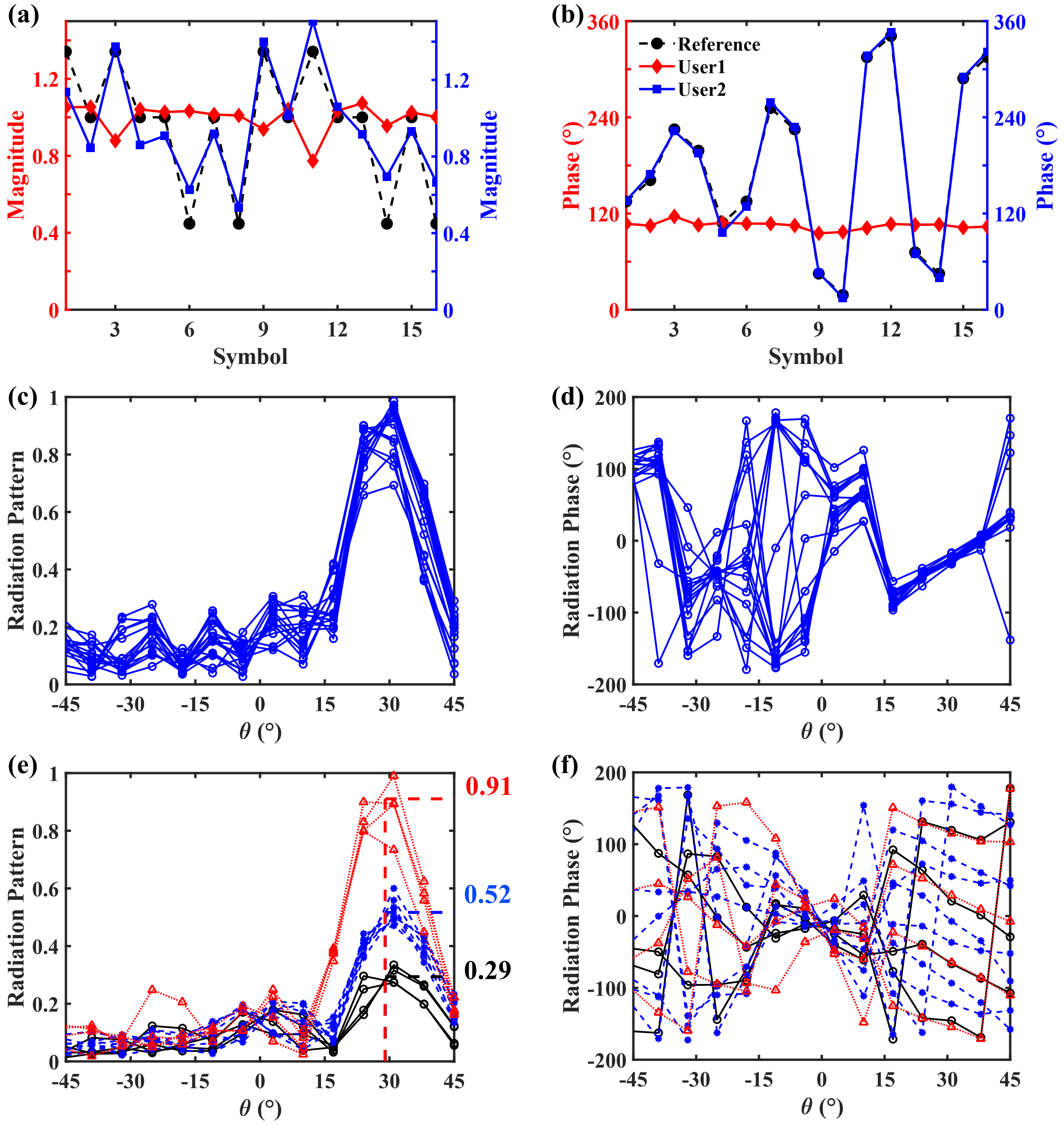

Figure S27. Results of the second group of tests for the dual-channel 16QAM. (**a**) The magnitudes and (**b**) phases of the signals in the desired directions. (**c**) The magnitudes and (**d**) phases of fields in the $\varphi = 0°$ plane. (**e**) The magnitudes and (**f**) phases of fields in the $\varphi = 90°$ plane.

Note 10. The distorted constellation diagrams in other directions

We have added the distorted constellation diagrams in other directions for each modulation scheme, as shown in Figure S28.

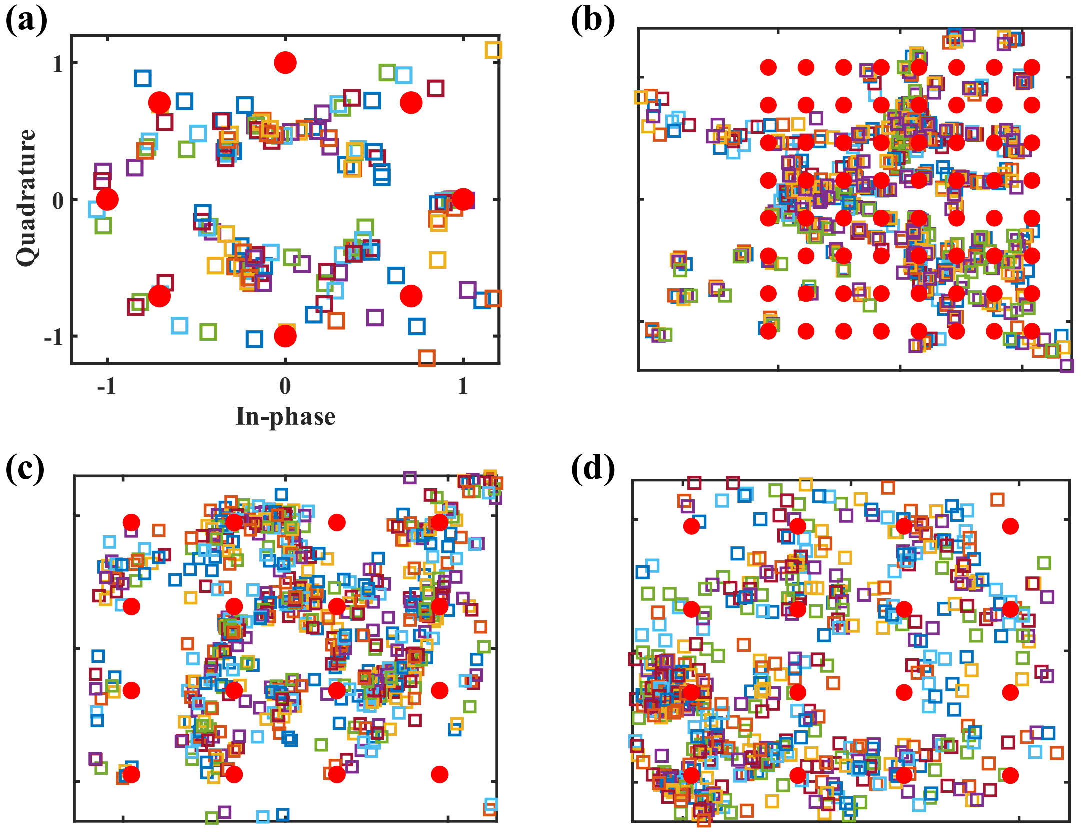

Figure S28. The distorted constellation diagrams in other directions for demonstrating the directional security. (**a**) The diagrams for the single-channel 8PSK experiment in the directions $(-45° \sim 29°)$ and $(-5° \sim 20°)$ in the $\varphi = 0°$ plane. (**b**) The diagrams for the single-channel 64QAM experiment in the directions $(-45° \sim 12°)$ and $(28° \sim 40°)$ in the $\varphi = 0°$ plane. (**c**) The diagrams for the double-channel 16QAM experiment in the directions $(-45° \sim 18°)$ and $(39° \sim 45°)$ in the $\varphi = 0°$ plane. (**d**) The diagrams for the double-channel 16QAM experiment in the directions $(-45° \sim 18°)$ and $(39° \sim 45°)$ in the $\varphi = 90°$ plane.

Note 11. The experiment for measuring the direction of desired users

The principle of sum- and difference- beam angle measurement is shown in Figure S29. The metasurface operates in receiving mode when the target user radiates incoming wave. Meanwhile, the metasurface scans in the whole space and generates sum, azimuth difference, and elevation difference beams respectively in three consecutive time slots by switching the coding sequences with a microcontroller unit (MCU). The power of sum beam $P_{\Sigma}(\theta,\ \varphi)$ reaches the maximal in the direction of the incoming wave. The power of difference beam $P_{\Delta}(\theta,\ \varphi)$ reaches the minimal in the direction of the incoming wave. We define the magnitude

radio of the power of the sum- and difference- beams as

$$r(\theta,\ \varphi) = \frac{P_{\Sigma}(\theta,\ \varphi)}{P_{\Delta}(\theta,\ \varphi)}. \tag{S19}$$

According to the physical meaning of $r(\theta,\ \varphi)$, the ratio reaches the maximal in the direction of the incoming wave. Therefore, we can plot the curve of $r(\theta,\ \varphi)$ and get the estimated direction $(\theta,\ \varphi)$ of the incoming wave.

To demonstrate the ability of measuring DOA of the incoming wave, we firstly conducted experiments to show that our programmable metasurface can generate the sum-, azimuth difference-, and elevation difference- beams with good performance. Wen then calculated the curve of $r(\theta,\ \varphi)$ using Equation (S29) and estimated the DOA of the incoming wave by finding the maximal $r(\theta,\ \varphi)$.

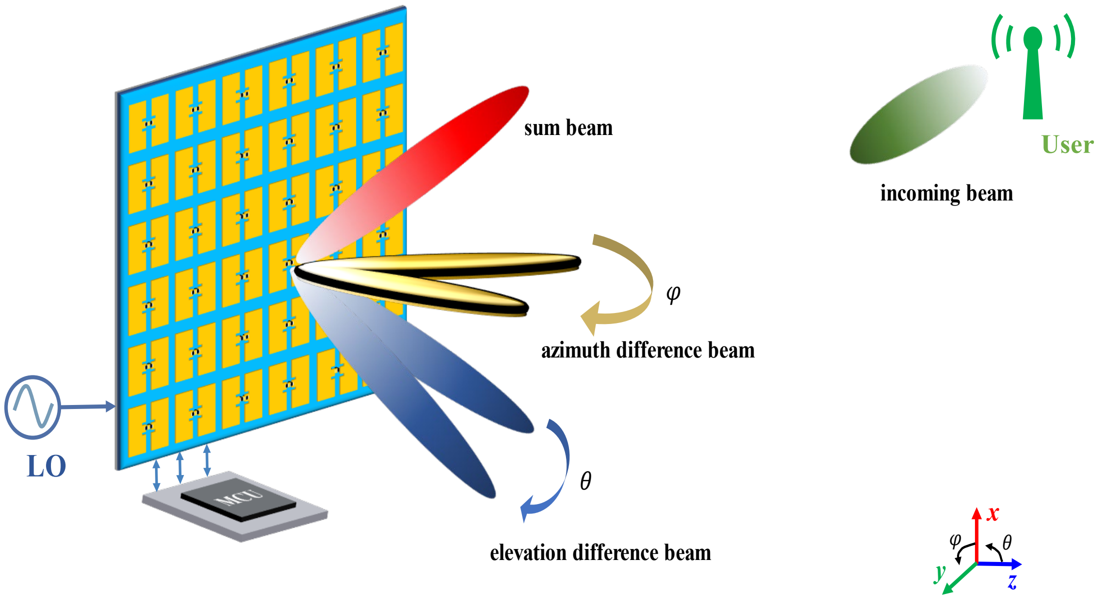

Figure S29. The conceptual diagram of sum- and difference- beam angle measurement.

1. The measured sum- and difference-beams

Figures S30a-S30c demonstrate the measured sum, azimuth, and elevation difference beams, respectively. The main-lobe angle of the sum beam is -1°. The null angles of the azimuth and elevation difference beams are 0° and -1°, respectively. The results show good agreement with

the excitation. As shown in Figure S31, the results of the sum beam and the azimuth difference beam is only given for the case of the direction of 30° due to the restriction of measurement conditions. However, the elevation difference beam can also be generated using the programmable metasurface.

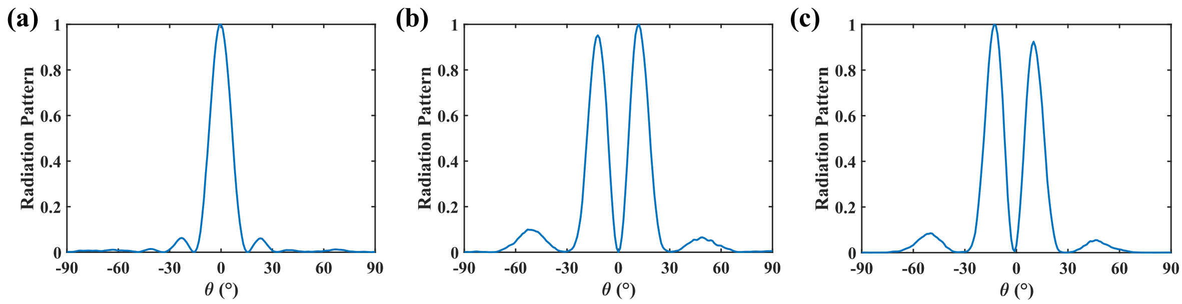

Figure S30. Measurements for the direction of 0°. (**a**) The sum beam. (**b**) The azimuth difference beam. (**c**) The elevation difference beam.

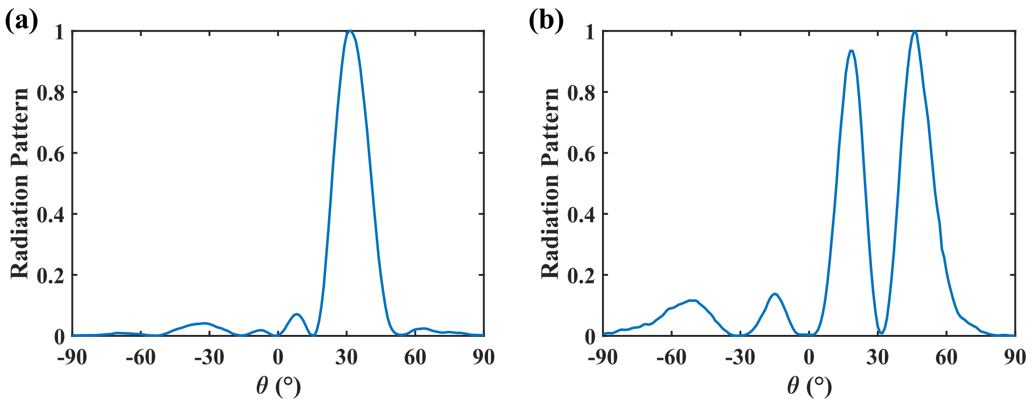

Figure S31. Measurements for the direction of 30°. (**a**) The sum beam; (**b**) The azimuth difference beam.

2. Measuring DOA of the incoming wave

The experimental scene for measuring DOA of the incoming wave is shown in Figure S32. The user equipped with the proposed metasurface can flexibly generate the incoming waves with different angles. The metasurface in the base station operates in receiving mode and scans in the whole space. During the scan, the metasurface generates sum, azimuth difference, and elevation difference beams respectively in three consecutive time slots by switching the coding sequences using MCU. It is worth noting that the beam scanning rate of the metasurface (about 1° per second) is much lower than the switching rate of MCU, thus the metasurface approximately obtains different data of magnitudes from the three beams simultaneously.

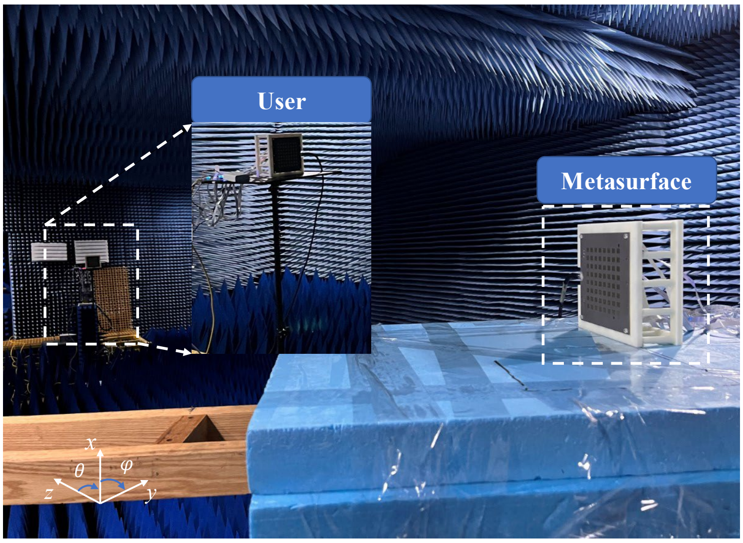

Figure S32. The photo of the experimental scene for measuring DOA of the incoming wave.

Figures S33a and S33b demonstrate the measured magnitude ratio in the azimuth and elevation planes. The directions of the maximal ratio in the azimuth and elevation planes are 0° and -1°, respectively. The estimated angle $(\theta,\ \varphi) = (0°,\ -1°)$ is in good agreement with the true direction of the incoming wave, which demonstrates the feasibility of our scheme. Figure S34 demonstrates the results for the incoming wave of 30°. The estimated angle is $\theta = 31°$, which matches well with the ground truth.

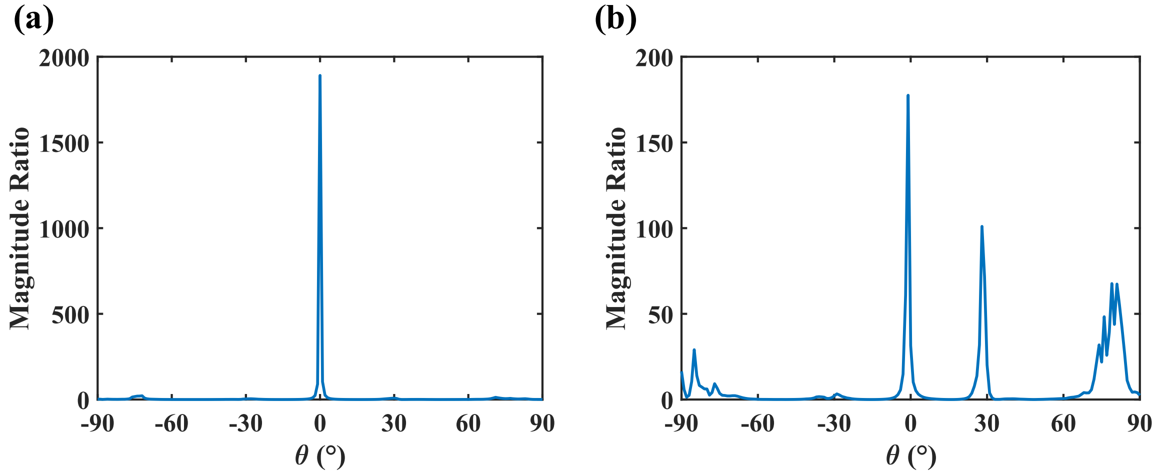

Figure S33. Magnitude ratio for the incoming wave of 0°. (**a**) The magnitude ratio of the sum and azimuth difference beam. (**b**) The magnitude ratio of the sum and elevation difference beam.

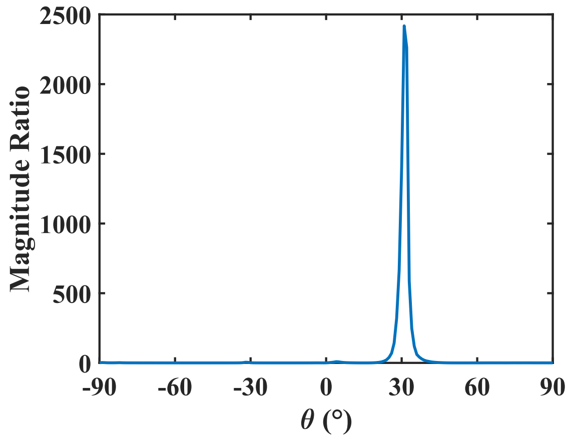

Figure S34. The magnitude ratio of the sum and azimuth difference beam for the incoming wave of 30˚.

Note 12. The transmission rate of the DIM scheme

The transmission rate limitation of the DIM scheme is mainly determined by the ON-OFF switch speed of meta-atoms and the modulation order. The switching speed of meta-atoms depends on the performances of adopted PIN diodes and the connector between the FPGA board and programmable metasurface. On the one hand, the adopted PIN diode (MACOMMADP-000907-14020x) can support a switching speed up to 2-3 ns. However, the switch speed of meta-atoms cannot reach the limit due to a relatively low-speed connector. Figure S35 demonstrates the measured control waveforms with different switching frequency using an oscilloscope. We can observe that the signal waveform with 50 MHz is relatively distorted due to the influence of the parasitic inductance of the connector. Therefore, the modulation frequency shift of the current DIM scheme can reach around 10 MHz.

Furthermore, we should emphasize that the current DIM scheme is a proof-of-concept DIM scheme demonstrating its flexibility for two-dimensional and multi-user directionally secure transmission using high-order modulation. The current transmission rate is 2 Mbps using 16QAM modulation. The limitation of the modulation speed of the DIM scheme can be largely mitigated by, e.g., (1) exploiting a high-speed connector (such as FMC, PCI Express); (2) packaging the FPGA and programmable metasurface on one PCB board; (3) optimizing the circuit layout after professional signal integrity analysis.

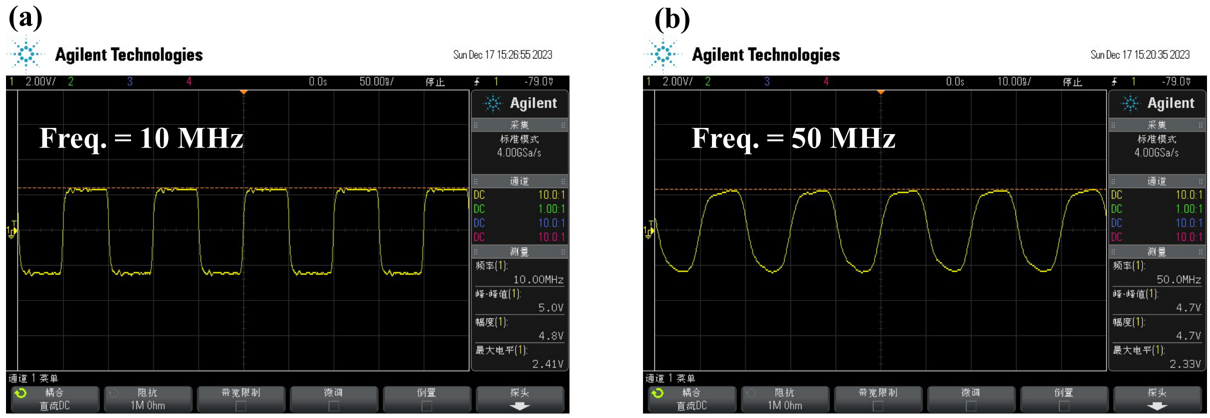

Figure S35. Measured control signal waveforms with different frequencies. (**a**) 10 MHz. (**b**) 50 MHz.

Note 13. The analysis of the secure zone

Here, we propose the concept of the secure zone, defined by the value of EVM, to separate the users and eavesdroppers. The initial step is to delineate the criteria, which involves the determination of the minimum angular width required to ensure directional security. Then the theoretical maximum number of supported channels is calculated according to the above angular width.

1. The minimum angular width to ensure directional security

As mentioned in the main text, the EVM is a critical metric to assess the similarity between received fields and reference constellation points. Particularly, the value of EVM is lower in the vicinity of desired users. In this context, we define the concept of the secure zone where the value of EVM is smaller than a certain threshold ε to separate the desired users and eavesdroppers. As shown in Figure S36, the EVM has a strong correlation with the radiation pattern of the metasurface, with lower value of EVM near the main lobe beam. Therefore, we utilize the main lobe beam as the secure zone and set the half-power beamwidth as the minimum angular width.

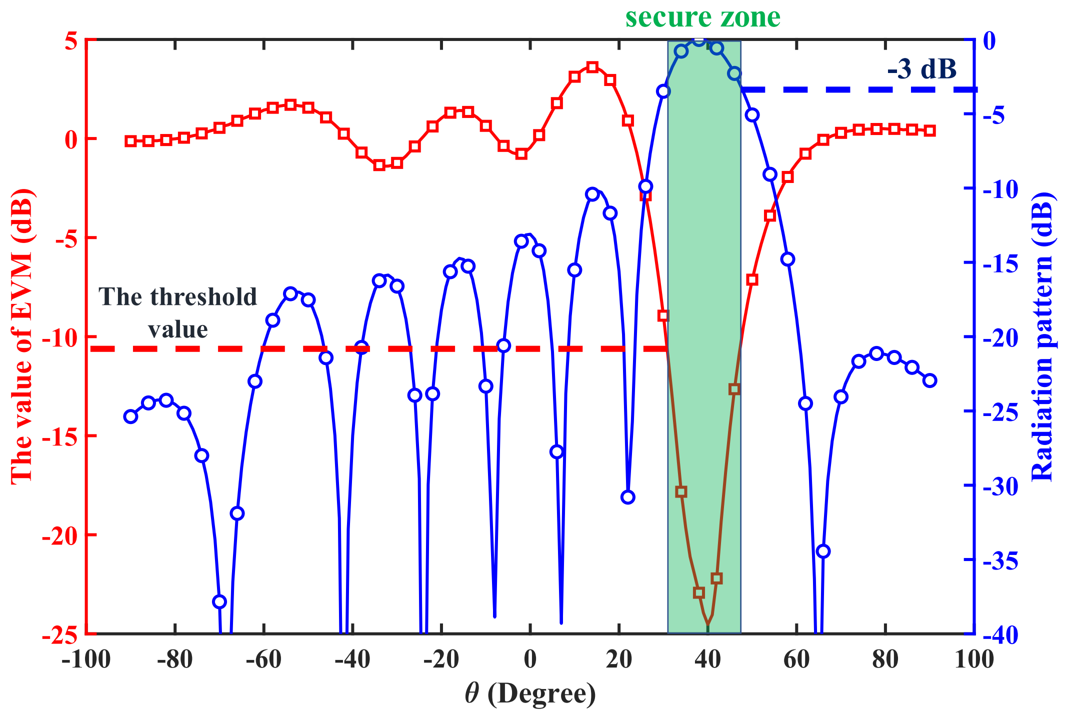

Figure S36. The relationship between EVM and the radiation pattern of the metasurface. The secure zone, marked by the transparent and green rectangle, is in the main lobe beam of the metasurface.

We firstly estimate the value of threshold ε when the secure zone is the main lobe beam. For simplicity, we here consider the one-dimensional situation. The definition of EVM is

$$EVM(\theta) = \sqrt{\frac{\frac{1}{N}\sum_{n=1}^{N} |y_n(\theta) - s_n|^2}{\frac{1}{N}\sum_{n=1}^{N} |s_n|^2}}, \tag{S20}$$

where y_n, s_n, and N are the received field, the reference constellation point, and the number of trials, respectively; y_n and s_n are normalized to ensure $\mathbb{E}[|y_n|^2] \approx 1/N\sum_{n=1}^{N}|y_n|^2 = 1$ and $\mathbb{E}[|s_n|^2] \approx 1/N\sum_{n=1}^{N}|s_n|^2 = 1$ when $N \to \infty$. The received field y_n can be decomposed into two parts, namely, $y_n(\theta) = f_n(\theta) + n$, where n includes the error caused by the quantization effects of meta-atoms and the additive white Gaussian noise. Therefore, the ideal field $f_n(\theta)$ is equal to s_n in the desired direction. In other directions, $f_n(\theta)$ is smaller than s_n (notice that the desired direction is in the main lobe beam). The value of EVM at the edge of main lobe beam is

$$
\begin{aligned}
EVM_{3\mathrm{dB}} &\approx \sqrt{\mathbb{E}[|y_n(\theta_{3dB})-s_n|^2]} \\
&= \sqrt{\mathbb{E}[|f_n(\theta_{3dB})-s_n|^2]+2\mathbb{E}[|f_n(\theta_{3dB})-s_n|\bullet|n|]+\mathbb{E}[|n|^2]} \\
&= \sqrt{\mathbb{E}[|f_n(\theta_{3dB})-s_n|^2]+\mathbb{E}[|n|^2]} \\
&\geq \sqrt{(\frac{\sqrt{2}}{2}-1)^2\mathbb{E}[|s_n|^2]} \\
&= 1-\frac{\sqrt{2}}{2} \approx -10.7\ \mathrm{dB}.
\end{aligned}
\tag{S21}
$$

In Equation (S21), we suppose that the signal is statistically independent with noise. After ignoring the noise, the maximum value of EVM in the main lobe beam is about -10.7 dB. We should emphasize that the above value is calculated under the assumption of infinite resolution. For the case of 2-bit phase quantization, we will give the value later.

To demonstrate the theorical estimation, we conduct simulations to calculate the half-power beamwidth (i.e., 3-dB width) of the radiation pattern and the secure zone width (i.e., the region where $EVM <= -10.7$ dB). According to the above theory, the two widths are almost equal. We here consider the single-channel DIM that supports 16QAM scheme, and the desired directions are $\theta = 10°$, 25°, 40°, and 55°, respectively. We also change the number of meta-atoms with 4, 8, and 16 to validate the scalability. Figures S37(a) and S37(b) demonstrate how the half-power beamwidth and the width of the secure zone varies with the number of meta-atoms. As indicated, the widths decrease as the number of meta-atoms increases. Specifically, when the number of meta-atoms doubles, the widths decrease by half, which indicates that a larger array has superior characteristics of directional security. The difference between the two widths is small and decrease as the number of meta-atoms increases, as shown in Figure S37(c). Taking the case of 8 meta-atoms as an illustrative example, we extract the detailed values of the widths and the difference between them, as listed in Table S4. The difference between the two widths is smaller than $1°$. It indicates that the two widths are almost equal, being consistent with the theoretical predication.

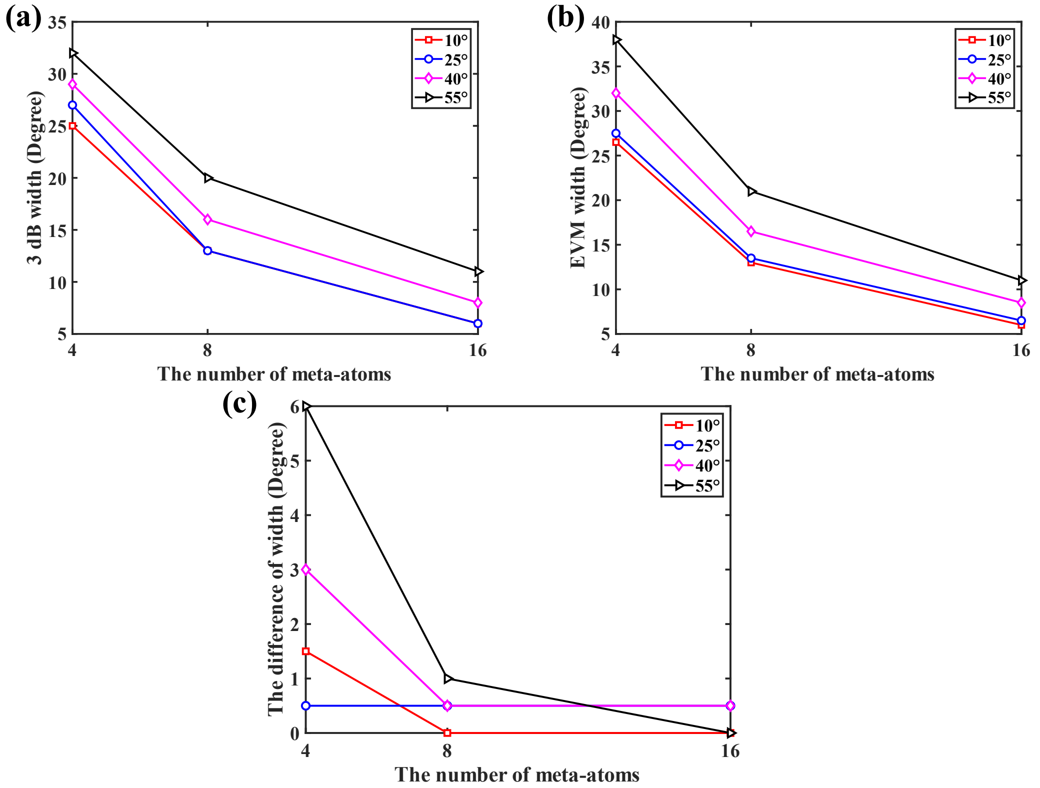

Figure S37. The simulation results to demonstrate the minimum angular width under the assumption of infinite resolution. (**a**) The half-power beamwidth of the radiation pattern. (**b**) The secure zone width (i.e., the region where $EVM <= -10.7$ dB). (**c**) The difference between the two widths.

Table S4. The detailed values when the number of meta-atoms is 8 in the infinite resolution.

width / direction	3-dB width	EVM width	The difference
10°	13°	13°	0°
25°	13°	13.5°	0.5°
40°	16°	16.5°	0.5°
55°	20°	21°	1°

The above theory and simulation demonstrate that the half-power beamwidth and the secure zone width (i.e., the region where $EVM <= -10.7$ dB) is the same under the assumption of infinite resolution. To find the secure zone width in the case of 2-bit phase quantization, we gradually increase the value of threshold ε until the difference between the two widths is minimal. As shown in Figure R38(a), the average difference between the two widths is smallest when the threshold value is $\varepsilon = -10.6$ dB. Figures S38(c) and S38(d) show the secure zone width (i.e., the region where $EVM <= -10.6$ dB) and the difference between the two widths, respectively. Taking the case of 8 meta-atoms as an illustrative example, we extract the detailed

values of the widths and the difference between them, as listed in Table S5. The difference of the two widths is smaller than 1.5°, which is the smallest in many attempts about the value of threshold ε.

Furthermore, we quantify the noise according the optimal threshold value. On the one hand, the value of EVM at the edge of main lobe beam in the case of 2-bit phase quantization is $EVM_{3\text{dB}} \approx \sqrt{\mathbb{E}[|f_n(\theta_{3dB}) - s_n|^2] + \mathbb{E}[|n|^2]} \approx -10.6$ dB. On the other hand, the value of EVM in the case of infinite resolution is $EVM_{3\text{dB}} \approx \sqrt{\mathbb{E}[|f_n(\theta_{3dB}) - s_n|^2]} \approx -10.7$ dB. The value of the noise is about $\sqrt{\mathbb{E}[|n|^2]} \approx -27$ dB. Hence, we can claim that, no matter how the scale of the metasurface increases, the minimum EVM in the desired direction is bigger than -27 dB under the constraint of 2-bit phase quantization. As shown in Figure S36, the value of EVM in the desired direction is about -24.8 dB, which is very close to the minimum and indicates the good performance of our discrete optimization algorithm.

If we utilize the main lobe beam of the metasurface as the secure zone and set the half-power beamwidth as the minimum angular width, the maximum value of EVM is -10.6 dB in the case of 2-bit phase quantization. The threshold value may be too large to be good enough to separate desired users and eavesdroppers, especially in the case of high-order QAM scheme. It is obviously that the secure zone width decreases if one reduces the threshold value ε. However, there will be no clear relationship between the secure zone width and the threshold value. More importantly, there will be also no clear relationship between the secure zone width and the scale of the metasurface. Therefore, we employ the main lobe beam of metasurface as the secure zone.

Table S5. The detailed values when the number of meta-atoms is 8 in the 2-bit phase quantization.

width / direction	3-dB width	EVM width	The difference
10°	13°	11.5°	-1.5°
25°	13°	14.5°	1.5°
40°	16°	16°	0°
55°	20°	19.5°	-0.5°

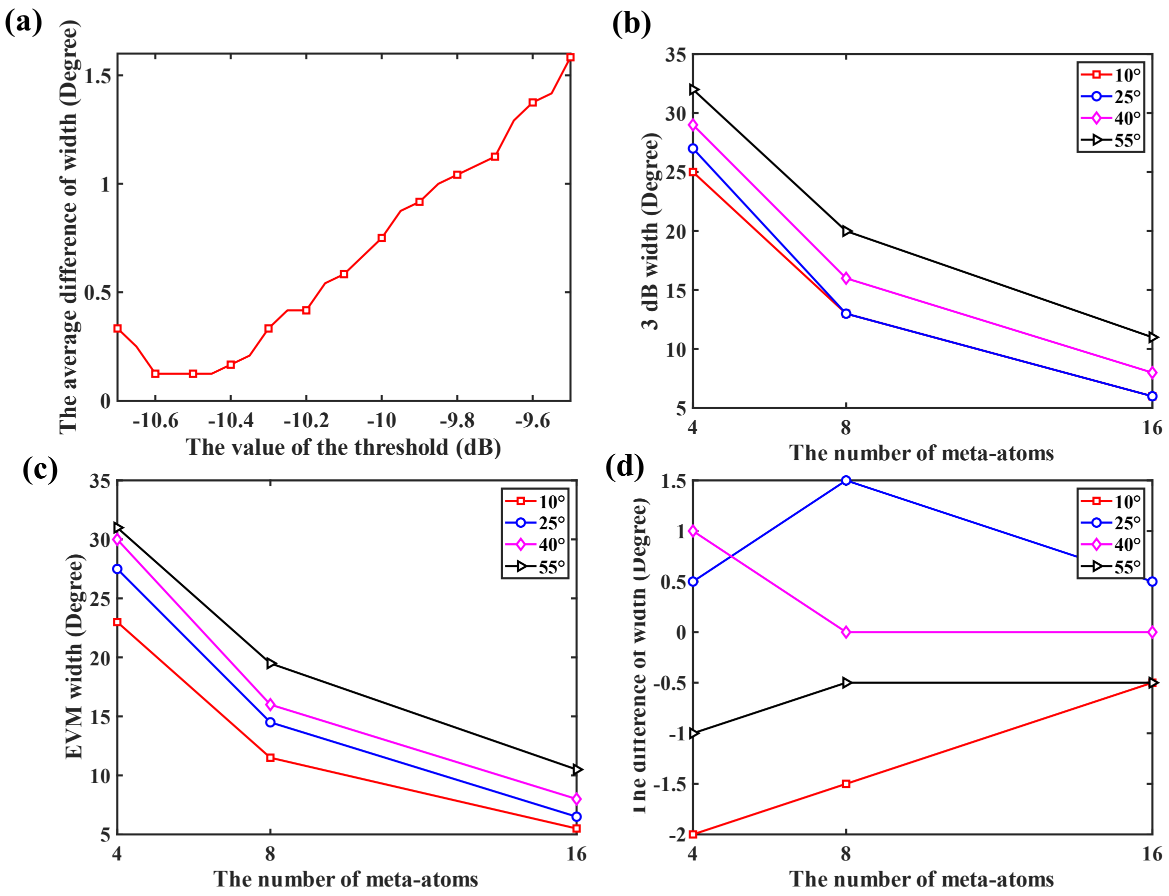

Figure S38. The simulation results to demonstrate the minimum angular width in the case of 2-bit phase quantization. (**a**) The average difference between the two widths as the threshold value changes. (**b**) The half-power beamwidth of the radiation pattern. (**c**) The secure zone width (i.e., the region where $EVM <= -10.6$ dB). (**d**) The difference between the two widths when the threshold value is -10.6 dB.

2. The maximal number of supported channels

If we utilize the main lobe beam of the metasurface as the secure zone, the minimum angular width has a closed form. For a metasurface with the aperture size A, the solid angle of the half-power beam is approximately written[16],

$$\Omega = \frac{\lambda^2}{A}, \tag{S22}$$

where λ is the wavelength. The maximal number of supported channels in the front-half plane is given by

$$N = \frac{2\pi}{\Omega} = \frac{D}{2}, \tag{S23}$$

where D is the directivity of the metasurface.

We should emphasize that Equations (S22) and (S23) ignore the issues of nonuniform surface current on the metasurface and the expansion of the half-power beamwidth under large-angle radiation. The actual number of supported channels will be smaller than the theoretical value $D/2$.

References

1. Y. Liu, J. Li, A. P. Petropulu, *IEEE Transactions on Information Forensics and Security.* **2013**, 8, 682.
2. Q. Wang, F. Zhou, R. Q. Hu, Y. Qian, *IEEE Transactions on Wireless Communications.* **2021**, 20, 2592.
3. R. M. Yamada, A. O. Steinhardt, L. Mili, *IEEE Transactions on Wireless Communications.* **2017**, 16, 8026.
4. T. A. Tsiftsis, C. Valagiannopoulos, H. Liu, A. A. A. Boulogeorgos, N. I. Miridakis, *IEEE Vehicular Technology Magazine.* **2022**, 17, 27.
5. D. Tulegenov, C. Valagiannopoulos, *Journal of Applied Physics.* **2022**, 131.
6. M. P. Daly, J. T. Bernhard, *IEEE Transactions on Antennas and Propagation*. **2009**, 57, 2633.
7. J. Y. Dai, W. Tang, L. X. Yang, X. Li, M. Z. Chen, J. C. Ke, Q. Cheng, S. Jin, T. J. Cui, *IEEE Transactions on Antennas and Propagation*. **2020**, 68, 1618.
8. M. Z. Chen, W. Tang, J. Y. Dai, J. C. Ke, L. Zhang, C. Zhang, J. Yang, L. Li, Q. Cheng, S. Jin, T. J. Cui, *National Science Review*. **2022**, 9, nwab134.
9. H. Zhao, Y. Shuang, M. Wei, T. J. Cui, P. D. Hougne, L. Li, *Nature Communications*. **2020**, 11, 3926.
10. X. Wan, C. Xiao, H. Huang, Q. Xiao, W. Xu, Y. Li, J. Eisenbeis, J. Wang, Z. Huang, Q. Cheng, S. Jin, T. Zwick, T. Cui, *Engineering*. **2022**, 8, 86.
11. J. Zhao, X. Yang, J. Y. Dai, Q. Cheng, X. Li, N. H. Qi, J. C. Ke, G. D. Bai, S. Liu, S. Jin, A. Alu, T. J. Cui, *National Science Review.* **2019**, 6, 231.
12. M. Wei, H. Zhao, Y. Chen, Z. Wang, T. J. Cui, L. Li, *Applied Physics Letters*. **2023**.

13. M. Wei, H. Zhao, V. Galdi, L. Li, T. J. Cui, *Nature Electronics*. 2023, 6, 610.

14. S. Jacobsson, G. Durisi, M. Coldrey, T. Goldstein, C. Studer, *IEEE Transactions on Communications*. **2017**, 65, 4670122.

15. S. SeyedinNavadeh, M. Milanizadeh, F. Zanetto, G. Ferrari, M. Sampietro, M. Sorel, D. A. B. Miller, A. Melloni, F. Morichetti, *Nature Photonics.* **2023**.

16. J. D. Kraus and R. J. Marhefka, *Antennas for All Applications*. New-York: McGraw-Hill, 2002.
